# The application of fNIRS in studies on occupational workload: a systematic review

**DOI:** 10.3389/fpubh.2025.1560605

**Published:** 2025-04-22

**Authors:** Robin Gemmerich, Ole Müller, Andrea Schaller

**Affiliations:** ^1^Department of Workplace Health Promotion and Prevention, University of the Bundeswehr Munich, Neubiberg, Germany; ^2^Agito Gesundheit GmbH, Cologne, Germany

**Keywords:** functional near-infrared spectroscopy, occupational workload, reporting, workplace health promotion, neuroimaging

## Abstract

**Background:**

Occupational workload can contribute to significant health problems such as chronic stress, fatigue and burnout. To investigate the underlying mechanisms, it is necessary to monitor brain activity in real work environments. Functional near-infrared spectroscopy (fNIRS) is a portable, non-invasive neuroimaging method that captures neural correlates of occupational workload under natural conditions. However, despite its increasing application, a comprehensive overview of fNIRS-based research in this field is lacking. Therefore, this systematic review examines how fNIRS can be utilized to investigate occupational workload.

**Methods:**

Following PRISMA 2020 guidelines, we conducted our systematic review by searching Web of Science, PubMed, and Scopus between November 15, 2023 and March 20, 2025. We included all studies published in English or German at any date, as long as they examined healthy adult professionals performing occupational tasks with functional near-infrared spectroscopy (fNIRS). Extracted data included study characteristics, workload details, signal processing methods, main fNIRS findings, and study quality, assessed using the JBI Critical Appraisal Tool.

**Results:**

We included 41 studies. Of these, 23 reported a significant increase in oxygenated hemoglobin (HbO) concentration and functional connectivity in the prefrontal cortex (PFC) under higher occupational workload conditions. Only five studies examined typical office tasks. Nine studies analyzed differences in cortical activation between experts and novices, with experts showing increased HbO concentration in the PFC than novices. Regarding methodology, 26 studies used standardized optode placements, while only 17 applied systemic and extracerebral artifact correction. Small sample sizes and the absence of randomized controlled trials limited the reliability and reproducibility of the findings.

**Conclusion:**

Functional near-infrared spectroscopy effectively detects neural correlates of occupational workload and provides objective insights into cognitive demands in real-world work settings. Standardizing optode placement, harmonizing signal-processing methods, and increasing sample sizes would enhance the validity and comparability of future research. Expanding investigations to typical office environments is also crucial for understanding daily workload and for developing interventions that promote employee well-being and productivity. Overall, fNIRS represents a promising tool for establishing evidence-based workplace health promotion strategies across diverse occupational settings.

## 1 Introduction

Occupational workload significantly impacts the well-being and performance of workers in industrialized nations (1). To effectively address this challenge, it is crucial to understand and accurately measure occupational workload. Several key theoretical frameworks have been developed to facilitate this understanding, including Cognitive Load Theory (2, 3), the concept of mental workload (4–6), the Job Demand-Control model (7), and the Effort-Reward Imbalance model (8). These theories conceptualize occupational workload as a multidimensional construct that encompasses cognitive, physical, and psychosocial demands, which in turn shape the available cognitive resources an individual can allocate to meet task demands (9). Whilst excessive workload decreases human performance, an overly low workload potentially reduces motivation and interest (10). Thus, the level of attentional resources required to meet both objective and subjective performance criteria can be influenced by task demands, external support, and previous experiences (8). Increasing human-computer interaction, globalization, and demographic changes in industrialized nations are widely recognized as factors that heighten pressure on employees (11–13). These challenges are intensified by constant availability, faster workflows, greater complexity in modern work environments, and often insecure job conditions, all of which increase pressure on employees (14). When occupational demands chronically exceed an individual’s capacity to cope, this sustained imbalance can lead to serious health issues such as exhaustion and burnout (15). These health issues have become major socio-economic challenges for companies (16), and impose a substantial burden on health care systems (17–19).

Traditionally, occupational workload has been assessed using standardized questionnaires like the NASA Task Load Index (20). Since physiological measurements allow for unobtrusive data collection without interfering with primary tasks, interest in these objective methods has been increasing (21). Techniques like electrocardiography (ECG), eye tracking, respiration, and electromyography (EMG) provide quantifiable insights but do not directly capture the underlying neural mechanisms of occupational workload (22, 23). To address this limitation, traditional neuroimaging methods such as electroencephalography (EEG) and functional magnetic resonance imaging (fMRI) have emerged as critical tools for assessing the neural correlates of occupational workload (9, 23–25). These methods offer objective insights that surpass traditional physiological measures, allowing for a more precise characterization of neuronal activity (26). Specifically, fNIRS facilitates the detection of neural overloads within executive function networks, which are critical for processes such as decision-making and attentional control (27, 28). Neural overload occurs when cognitive demands exceed the brain’s processing capacity, particularly in executive functions (29, 30). This overload leads to heightened activity in regions like the dorsolateral prefrontal cortex (DLPFC), anterior cingulate cortex (ACC), and inferior parietal lobule (IPL), leading to cognitive impairment, reduced performance, and increased error rates in work contexts (31–33). If this neural overload remains unaddressed, it can contribute to long-term health issues like chronic stress, fatigue, and burnout (34, 35). However, while effective for neural insights, EEG and fMRI are often unsuitable for dynamic work environments due to their susceptibility to movement artifacts from head and body movements and the requirement for participant immobility (36–38). Continuous monitoring of neural activity in real-world occupational settings is essential for understanding how workload accumulates and impacts cognitive performance, enabling interventions to prevent cognitive overload (39).

Functional near-infrared spectroscopy (fNIRS) is considered a promising tool for measuring occupational workload, as it provides robust data under ecologically realistic conditions (40–45). In brief, fNIRS is an imaging technique indirectly measuring brain activity by using near-infrared light (650–1,000 nm) absorbed by hemoglobin in the brain to measure changes in the concentrations of oxygenated hemoglobin (HbO) and deoxygenated hemoglobin (HbR) (46). Compared to other imaging techniques such as fMRI and EEG, fNIRS offers moderate spatial resolution and potentially better temporal resolution but is limited to neocortical regions (47), and exhibits a lower signal-to-noise ratio than fMRI (48). FNIRS advantages include tolerance for motion artifacts, ease of use, portability, and low costs, making it suitable for studies in natural settings and promoting its use in real work environments (47, 49).

Due to its practical feasibility and advantages over other imaging techniques, fNIRS has increasingly been employed to assess occupational workload in real-world settings such as piloting aircraft (40), operating urban rail transport systems (50), or performing office work (51). These applications demonstrate the potential of fNIRS to provide valuable neurophysiological insights in occupational environments. By identifying neural markers under everyday conditions, fNIRS offers objective data crucial for the early detection and prevention of occupational overload and long-term health issues like chronic stress, fatigue, and burnout. This technology bridges the gap between basic neuroscience research and occupational health promotion, providing a practical tool for implementing evidence-based interventions in the workplace. Therefore, this systematic review addresses the following research questions: How is fNIRS utilized to investigate occupational workload?

## 2 Materials and methods

This systematic review is conducted according to the Preferred Reporting Items for Systematic Reviews and Meta-Analysis Extension for Systematic Reviews PRISMA-2020 guidelines (52, 53).

### 2.1 Search strategy

A systematic literature search was initially conducted from November 15, 2023, to December 01, 2023, in the electronic databases PubMed, Web of Science, and Scopus. To ensure that the review reflected the most recent state of research, an updated search was performed from December 1, 2023, to March 20, 2025. Eight additional studies identified during this second screening were assessed for eligibility and fully integrated into the review. These studies are also listed in Supplementary Table S2. Search queries systematically integrated pertinent keywords using Boolean operators (‘OR’, ‘AND’) to optimize precision. A detailed description of these queries is provided in Table 1.

**Table 1:** Search terms utilized in the databases PubMed, Web of Science, Scopus for the measurement of health hazards at the workplace using fNIRS Boolean operator.

Boolean operator	Search terms
AND	(“Spectroscopy, Near-Infrared OR “functional near-infra*” OR “fNIRS”) (“Occupational Groups” OR “Occupation*” OR “Work” OR “Workplace” OR “Employment” OR “Occupational Health” OR “Occupational Diseases” OR “Occupational Stress” OR” Stress*” OR “Workplace Health*” OR “Workload” OR “Ergonomics” OR “Neuronal Plasticity”)

### 2.2 Inclusion criteria

The eligibility criteria were guided by the PICOS guidelines (54, 55), as shown in Table 2. We used a combined keyword strategy instead of separate terms for each PICO component to ensure a broad capture of studies using fNIRS in occupational contexts. This approach helped identify a wide range of articles without excluding those that might use different terminology for similar research questions. The inclusion criteria included articles that were: (a) written in English and / or German, (b) based on measurements of cortical activity using functional Near-Infrared Spectroscopy (fNIRS), (c) involving healthy working adults studied within their professional milieu, and (d) designed with a workplace-oriented experimental focus, specifically addressing professional and occupational contexts.

**Table 2:** PICOS criteria for study inclusion.

PICOS	Inclusion criteria
Population (P)	Healthy working individuals studied within their professional milieu of all ages and gender.
Intervention (I)	Measurement of cortical activity using fNIRS during health-related workplace exposure
Comparison (C)	With or without a control group.
Outcomes (O)	Overview of fNIRS applications in the context of mental workload in a working environment
Study type (S)	Experimental research design

### 2.3 Exclusion criteria

Studies that did not fulfill one of the following criteria were excluded from the literature search: (a) fNIRS was not used as the main modality for cerebral examination techniques; (b) lack of a clear occupational context, meaning that there was no direct association with job affiliation or occupational activity; and (c) study population of professional athletes and professional musicians. Professional athletes and musicians were excluded due to their unique performance contexts and specialized training demands, which differ substantially from conventional workplace tasks and could introduce confounding factors when interpreting fNIRS data in an occupational setting.

### 2.4 Study selection

The screening procedure utilized *Rayyan software* (56) to automatically eliminate duplicates from the collected literature. Subsequently, the titles and abstracts were evaluated based on the predefined inclusion and exclusion criteria and classified as relevant, irrelevant, or indeterminate. Records deemed irrelevant were discarded. The remaining articles underwent a full-text review for further evaluation and potential exclusion.

This entire process was executed in a double-blinded manner with two independent reviewers (RG, OM), which aimed to minimize the risk of biased outcomes and proactively address any methodological issues. Disagreements between the reviewers were resolved either by reaching a consensus or consulting a third reviewer (AS).

### 2.5 Data extraction

During the data extraction process, relevant study characteristics were collected with a meticulously designed data extraction sheet. These characteristics comprised the name of the first author and the publication year, the study sample, including occupation description, years of professional experience, sample size, sex, and age (as illustrated in Table 3). Additionally, aspects of the study design were examined using a second structured form, which encompassed specifications of the control group, procedures for randomization, protocols for follow-up, descriptions of conflict tasks, and types of workplace environments, as presented in Table 4.

**Table 3:** Study characteristics, participant demographics and occupational workload information of included studies.

First author (Year)	Participant demographics			Work experience and conditions		
	Sample size	Mean age (*SD*)	Occupation	Professional experience (Years/Hours)	Type of workload	Workplace environment type
Abujelala et al. (60)	31 local firefighters	*M* = 30.74 years (± 4.19)	Firefighter	6.9 years, ± 3.99	Mental workload under time pressure	Simulations with VR-scenarios
Ahlstrom (83)	3 Display symbology groups (D1-D3); 8 D1 subjects, 8 D2 subjects, 8 D3 subjects, Total: 24 participants	D1: *M* = 64, D2: *M* = 56, D3: *M* = 53	Pilots	Experience in flight hours. D1: M = 3,500, D2: *M* = 3,100, D3: *M* = 4,000	Mental workload	Simulation
Aksoy et al. (71)	22 (4f, 18 m)	*M* = 29.45 (± 2.4)	Surgery residents	5 months to 6 years, with a mean (SD) of 3.11 (1.72) year	Cognitive and mental workload	Simulation of Laparoscopic surgery (LapVR simulator) vs. Robot Assisted Surgery (RAS) (The Da Vinci Surgical System Si console)
Alyan et al. (76)	23 office workers	*M* = 28.6 (± 3.4)	Office worker	N/A	Occupational stress	Simulation
Alyan et al. (76)	10 office workers	*M* = 29.1 years (± 2.6)	Workers at workstation	N/A	Mental workload	Simulation
Andreu-Perez et al. (75)	12 novices, 11 trainees, 9 experts	Novices: *M* = 22.4 years (± 1.6), trainees: *M* = 33.8 years (± 2.9), experts: *M* = 42.7 years, (± 3.6)	Surgeon	In cases involving laparoscopic suturing: novices: 0, trainees: < 50, experts: > 50	Cognitive workload	Simulation in a box trainer (i-SIM, iSurgicals, UK)
Ayaz et al. (80)	24 certified professional controllers	Ages of 24 to 55	Air traffic control	3 to 30 years	Mental workload	Simulation in control workstations with high resolution radarscope
Chong et al. (77)	19 nurses, 19 students	Nurses: *M* = 30.44 (± 3.20), students: *M* = 20.68 (± 0.82)	Nurse	Nurses: (8.32 years working experience, ± 3.04), students: (2.70 years internship experience, ± 0.41)	Mental workload while cognitive distraction	Simulation
Fan and Yang (70)	20 experienced (20 m), 20 inexperienced (2f, 18 m), Total: 40 participants (2f, 38 m)	Experienced: *M* = 44.6, inexperienced: *M* = 25	Seafarer	Experienced: 17.78 years; inexperienced: 2.27 years	Mental workload	Simulation
Fan et al. (69)	20 experienced (1f, 19 m), 20 inexperienced (2f, 18 m), Total: 40 participants (3f, 37 m)	Experienced: *M* = 44.6 (± 15.5), inexperienced: *M* = 25 (± 5.4)	Seafarer	Experienced: (17.78 years, ± 15.73), inexperienced: (2.27 years, ± 2.54)	Decision-making	Simulation
Fan et al. (63)	10 professional watchkeeping officers	*M* = 35.1 (± 15.6)	Watchkeeping officers	N/A	Decision-making	Simulation
Harrison et al. (74)	12 air traffic control operators	N/A	Air traffic control operator	N/A	Cognitive workload	Simulation
Isbilir et al. (81)	8 experts, 6 novices	Experts: *M* = 30.5 (± 3.9), novices: *M* = 27.3 (± 2.1), Total: *M* = 28.9(± 3.6)	Operators of military land platforms	N/A	Cognitive workload	Simulation/training version of a military land platform (Roketsan Inc.)
Kakehashi et al. (86)	12 (6f, 6 m)	20–39 years old	Office workers	N/A	Cognitive workload	Simulation
Kawaguchi et al. (64)	6 airline captains, 6 trainee pilots	Airline captains: *M* = 48 (± 4), trainee pilots: *M* = 24 (± 2)	Pilots	Airline captains: 120 h (±72 h), trainee pilots 10,490 h (±1,260 h)	Mental workload	Simulation: Boeing 767–300 full-motion simulator
Le et al. (41)	5 (1f,4 m)	*M* = 38 years (± 10)	Driving	N/A	Mental workload while cognitive distraction	Real driving on a test course
Li et al. (50)	10 drivers (2f, 8 m)	*M* = 25 (± 3.0)	Metro driver	>1 year driving experience	Mental workload	Simulation
Li et al. (92)	18 (4f, 14 m)	23–29 years	Air traffic control	N/A	Decision-making	Simulation
Li et al. (90)	15 Intervention (f4,11m), 15 Control (f5,10m)	Intervention: *M* = 23.93 (± 0.96), Control: *M* = 23.86 (± 1.24)	Orthopedic surgeon	Postgraduate year 1–3: Intervention: PGY1: 14, PGY2: 1, Control: PGY1: 14, PGY3: 1	Workload under time pressure / stress	Simulation via VirtaMed ArthroS
Liu et al. (72)	24 R&D group (f1, 23 m), 26 sales group (1f, 25 m)	R&D group: *M* = 32.21 (± 6.04); sales group: *M* = 33.77 (± 6.12)	Employees within high-tech enterprises	Maintain their current positions for over a year	Occupational stress	Simulation
Menda et al. (79)	6 chase view subjects, 5 onboard camera interface subjects, Total: 11 laboratory personnel	N/A	Unmanned aerial vehicle operator	N/A	Cognitive workload and situational awareness	Simulation
Midha et al. (51)	18 office workers (8f, 10 m)	*M* = 31 (± 9.57)	Office work	N/A	Mental workload	Simulation
Modi et al. (68)	15 junior residents (PGY1-2), 8 intermediate residents (PGY3-4), 10 senior residents (PGY5), Total: 33 residents (6f, 27 m)	*M* = 33	Surgical residents	PGY1-2: 1–2 years’ experience, PGY3-4: 3–4 years’ experience, PGY5: 5 years’ experience	Mental workload under temporal stress	Simulation in a box trainer (i-SIM, iSurgicals, UK)
Modi et al. (65)	33 surgical residents (6f, 27 m)	*M* = 33 years; range, 29–56 years	Surgical residents	Experience > postgraduate year 1	Workload under temporal stress	Simulation in surgery simulator
Mora et al. (66)	26 organic farm, 22 conventional farm, Total: 48 (2f, 46 m);	25 farmers >35 years old; 23 farmers <35 years old	Farmworker	24 farmers >20 years’ experience; 24 farmers <20 years’ experience	Cognitive workload	Real
Naik et al. (91)	10 surgical residents (4f, 6 m)	N/A	Surgical residents	8.15 years	Cognitive workload	Simulation in Olympus EVIS EXERA III/VISERA box trainer
Palzes et al. (45)	26 organic farm, 22 conventional farm, Total: 48 participants (2f, 46 m)	Mean age not reported; 21 farmers: 18–29 years old, 13 farmers: 30–49 years old, 14 farmers: >50 years old	Farmworker	19 farmers: <14 years in agriculture, 14 farmers: 15–29 years in agriculture, 15 farmers: >30 years in agriculture	Working memory	Real
Pooladvand and Hasanzadeh (88)	33 (11f, 22 m)	N/A	Electrical line workers	1.5 years’ experience in construction industry	Decision-making, safety performance in stressful situation	Simulation
Pooladvand et al. (89)	33 (11f, 22 m)	N/A	Electrical line workers	1.5 years’ experience in construction industry	Mental workload under time pressure	Simulation
Singh et al. (67)	8 surgeons (1f, 7 m)	*M* = 34.5 (± 2.9)	Surgeon	N/A	Mental workload under time pressure	Simulation
Sun et al. (73)	16 novice teachers [NT (13f, 3 m)]; 18 expert teachers [ET (14f, 4 m)] and 34 students (27f, 7 m)	NT: *M* = 25.81 years (± 4.69); ET: *M* = 38.00 years (± 4.30) and students: *M* = 20.15 years (± 1.67)	Teaching	NT: (2.08 years, ± 2.16), ET: (16.14 years, ± 5.07)	Effects of teacher type on cooperative performance	Real
Tang et al. (61)	45 experienced pilots, 68 experienced drivers, 41 trainee pilots	Experienced drivers: *M* = 23.4 years (± 3.3); trainee pilots: *M* = 21.1 years (± 2.1)	Remotely operated vehicle operators	Pilots: 15 years; experienced drivers: N/A; trainee pilots: N/A	Mental workload	Simulation
Tian et al. (78)	80 engaged in unsafe behaviors (EUB), 26 not engaged in unsafe behaviors (NUB), Total: 106 (106 m)	NUB: *M* = 34.89 (± 6.77), EUB: *M* = 36.38 (± 6.40), Total: *M* = 27.3 (± 5.7);	Coal mine worker	NUB: (9.00 years, ± 7.06), EUB: (9.76 years, ± 7.02), Total: N/A	Mental fatigue	Real
Tian et al. (43)	17 morning shift, 18 afternoon shift, 19 night shift, total: 54	Morning shift: *M* = 36.8 years (± 6.77), afternoon shift: *M* = 37.5 years (± 7.61), night shift: *M* = 34 years (± 7.13), total: *M* = 36.06 years (± 7.42)	Coal mine worker	Morning shift: (10.29 years, ± 6.72), afternoon shift: (11.9 years, ± 8.39), night shift: (7.68 years, ± 7.33), total: (9.91 years, ± 7.81)	Cognitive workload	Real
Tian et al. (85)	89 coalmine workers	Group 1: *M* = 32. (± 4.38) Group 2: *M* = 32 (± 4.46) Group 3: *M* = 33 (± 5.33) Group 4: *M* = 32 (± 4.48)	Coal mine worker	N/A	Working memory	Simulation
Tyagi et al. (62)	17 stresses; 17 controls, Total: 34 (34 m)	stress: *M* = 29.76 (± 4.25); control: *M* = 31.71 (± 4.01),	Firefighter	Stress: (6.51 years, ± 3.95); control: (7.29 years, ± 4.1),	Mental workload under time pressure	Simulation
Verdière et al. (40)	12 pilots (1f, 11 m)	*M* = 24 years (± 3)	Pilot	N/A	Cognitive workload	Simulation in an Airbus A320 full motion simulator
Xu et al. (82)	13 (year 1 residents), 5 (year 2 residents), 7 (year 3 residents), 8 physicians, Total: 33 participants	Y1 residents: (*M* = 29.54, SD = 2.47), Y2 residents: (*M* = 29.20, SD = 1.64), Y3 residents: (*M* = 30.14, SD = 0.69), physicians: (*M* = 42.88, SD = 6.08)	Anaesthesiologist residents	Y1 residents: (0 years clinical experience), Y2 residents: (0 years clinical experience), Y3 residents: (0 years clinical experience), physicians: (9.88 years clinical experience, SD = 4.02)	Cognitive workload	Simulation
Zhang et al. (93)	25 Pilots (25 m)	Not reported; between 21 and 30 years old	Pilots	Experience in flight hours: 230 to 250 h	Mental workload	Simulation
Zheng et al. (87)	4 teacher (2f, 2 m), 60 students (30f, 30 m)	Teacher: M = 25 (± 2.4), students: M = 23 (± 2.3)	Teaching	Teacher: 6–7 years; students: N/A	Effects of teacher type on arithmetic Test	Simulation
Zheng et al. (42)	60 students (30f, 30 m), 4 teachers (2f, 2 m)	Teacher: M = 25 years (± 2.4); students: *M* = 23 years (± 2.3)	Teaching	Teacher: 6–7 years; students: N/A	Interaction quality and affiliation bond between students and teacher	Real

f, female; m, male; N/A, Not Applicable; VR, Virtual Reality.

**Table 4:** Study design aspects.

First author (Year)	Control conditions	Randomization	Follow-up	Brain regions-of-interest	fNIRS device	Performance evaluation methods and cognitive task protocols
Abujelala et al. (60)	Control group and stress group	No	No	dlPFC, mPFC, premotor cortex, SMA	NIRSport 2	VR-based training for firefighters: (1) Familiarization: 3 pipe-maintenance operations with/without stressors. (2) Training: 8 operations with/without stressors. (3) Buffer: no task. (4) Evaluation: 8 operations with/without stressors. Stressors: fires, alarms, smoke.
Ahlstrom (83)	None	Randomized order of conditions	No	PFC	NIR Smart fNIRS	Flight simulator route: Three groups (D1, D2, D3) with different colors and shapes of the cockpit symbols, weather avoidance without ATC responses with Cessna 172 simulator
Aksoy et al. (71)	None	Randomized order of conditions	No	PFC	fNIR device model 2,000 M (Devices LLC,)	Simulated surgical environments: two Blocks; RAS simulator in block 1 and the laparoscopy simulator in block 2. Both simulated within VR setting
Alyan et al. (76)	None	No	No	PFC	OT-R40	MIST: three random integers (0–99) with random operators, 90% estimated avg. time constraint. 20s rest, 30s MAT, 10 rounds. Two sessions, one per workstation type.
Alyan et al. (76)	None	No	No	dlPFC, VPC, FPC, OFC	OT-R40	MIST. three random integers (0–99) with random operators, 90% avg. time constraint. 20s rest, 40s task, 6 rounds. Two sessions, one per workstation type.
Andreu-Perez et al. (75)	None	No	No	PFC, MC, premotor cortex, SMA,	ETG-4000	Operator skill level during laparoscopic surgery: simulated laparoscopic suturing in a box trainer (iSim2; iSurgicals), performed three times. Subtasks: (1) Needle insertion, (2) Double-throw knot tying, (3) Single-throw knot tying.
Ayaz et al. (80)	None	Pseudorandom order of communication condition	No	dlPFC	fNIR device model 1,000 (Devices LLC,)	Two tasks: (1) Visual identity n-back (0–3). (2) ATC tasks: controlling simulated air traffic, voice/data communication with pilots. Task difficulty: 6, 12, 18 airplanes per sector.
Chong et al. (77)	None	No	Yes	PFC	OT-R40	Nurses under emotional states: Task with auditory stimuli (neutral and emotional). Five task/rest periods (60s each). Answering nursery questions in 60s. Second session after 6 weeks with different stimuli.
Fan and Yang (70)	None	No	No	dlPFC	NIRSport 88	Watchkeeping task on open sea. Spot ship, press button (20 min). Decision-making (5 min): monitor ship, assess collision risk, change course if needed. Distraction: report own coordinates.
Fan et al. (69)	None	Quasi-randomized group for distraction task	No	PFC	NIRSport 88	Watchkeeping on open sea, 180° view. Spot ship, press button. Decision-making: monitor ship, assess collision risk, change course. Distraction: report coordinates.
Fan et al. (63)	None	Randomized order of conditions	No	PFC, Broca Areal	NIRSport 2	Ship collision avoidance during watchkeeping during two conditions (low and high) workload. Participants works together in groups of two. High workload added several small recreational boats on radar
Harrison et al. (74)	None	Pseudorandom participant rotation	Yes	PFC	fNIR device model 1,000 (Devices LLC,)	Cognitive workload in aviation: Impact of CRA on ATCS over 3 days, 9 sessions. Three conditions: (1) No CRA. (2) Data-side with CRA. (3) Both with CRA. 3 test and 3 practice sessions/day.
Isbilir et al. (81)	None	No	No	PFC	fNIRS imager 1,002	Military land platform simulation (Roketsan Inc.). Two tasks: (1) Standard target engagement (data entry, initialization, checks, monitoring, engagement). (2) Engage two targets, second slightly out of range, trigger error, wait 60s, explain error.
Kakehashi et al. (86)	None	No	No	PFC	LIGHTNIRS	Office work efficiency: Two tasks, four lighting conditions. Arithmetic task: Modified Uchida-Kraeplin test, adding numerals in line, 30s, 3 trials. Copying task: Copying sentences in Japanese and English, 4 trials.
Kawaguchi et al. (64)	None	No	No	dlPFC	LIGHTNIRS	Five landings in a flight simulator: two in low and three in high maneuvers. During the high maneuvers, the weather was bad and the flight controller function was switched off. Before first session: 30-min training session to familiarize
Le et al. (41)	None	No	No	dlPFC	ASTEM’s fNIRS Astem Corp., Fukuoka, Japan	Driving on a test course, 60 experiments (following a car, autocross) at 40 km/h. 3 stages: driving only, driving +1-back task, driving +2-back task. Cognitive Task: 2-level n-back auditory digit recall task.
Li et al. (50)	None	No	No	PFC	WOT-100 system	Urban rail drivers: 3 test parts: determination tests, 70 min simulated driving, and determination test. Four driving tasks: (1) Pedestrian-vehicle scenario after 10 min. (2) Normal driving (20 min) with n-back multi-task (*n* = 3). (3) Repeat task 2. (4) Repeat task 2.
Li et al. (92)	None	No	Yes	PFC, MC, OC	NIR smart fNIRS	Task in real-time ATC: Three stages: Rest-stage (10 min), Task 1-stage (descriptive map, 10 min), Task 2-stage (optimized map, 10 min). Relaxation: 10 min between stages. Cognitive Task: n-back task (*n* = 2) over 5 days.
Li et al. (90)	Control group with no control condition	Participants were randomly divided into intervention and control group	Yes	PFC	NIRSIT	Pre-test: Knee Diagnostics & Loose Body Removal (simulator). Randomization: intervention (2-week mindfulness) vs. control (no training). Retraining: day 7. Post-test: day 14.
Liu et al. (72)	None	No	No	PFC	BS-3000	Verbal fluency test (VFT): a 30 s pre-task baseline, a 60 s task period, and a subsequent 60 s post-task baseline.
Menda et al. (79)	Onboard camera interface as control group	No	Yes	PFC	N/A	UAV operator workload and situational awareness: Seven flight sessions. Three performance goals: (1) Fly through test environment, maintaining safe distance from walls/obstacles. (2) Fly in correct directions around obstacles. (3) Fly over color targets in specified order. Duration: 2 h/day for 9 days.
Midha et al. (51)	None	No	No	PFC	Octamon	3 conditions, 5 min each. Reading tasks: Easy: basic materials. Medium: unread academic article. Hard: medium + secondary task. Writing tasks: Easy: describe tasks. Medium: outline availability and research. Hard: medium + say “blah” while writing
Modi et al. (68)	None	Randomized order of conditions	No	PFC	ETG-4000	Suturing on box trainer (iSim2). Two conditions: (1) Self-paced. (2) 2-min time limit per knot. Task: double throw, two single throws, 5 times each.
Modi et al. (65)	None	Randomized order of conditions	No	PFC	ETG-4000	Intracorporeal laparoscopic suturing on a box trainer (iSim2; iSurgicals). Two stages: (1) Self-paced surgery without time restriction. (2) Surgery under time pressure with a 2-min limit per knot.
Mora et al. (66)	Organic farm as control group	No	Yes	PFC, dlPFC, Broca Areal	NIRSport	Neurobiological effects of pesticide exposure: Three tasks: (1) Sternberg working memory (30 trials, list of 8 letters, determine if letter was in previous list). (2) Go/No-Go task (press button for non-X, withhold for X). (3) Wisconsin Card Sort (match cards based on unstated rule).
Naik et al. (91)	None	Randomized order of conditions	No	PFC	Artinis Brite24	Participants performed a standardized laparoscopic procedure (start to finish) with a consistent human assistant while completing a modified auditory N-back task at varying difficulty levels.
Palzes et al. (45)	Organic farm as control group	No	No	dlPFC	NIRSport	Sternberg letter-retrieval task (30 trials). List of 8 letters for 2 s, then determine if a single letter was in the previous list.
Pooladvand and Hasanzadeh (88)	None	No	No	PFC, SMA, primary motor cortex	Brite MKIII fNIRS	Electrical task under three conditions: (1) Normal conditions. (2) Time pressure. (3) Time pressure + secondary 2-back task (respond to number sequence).
Pooladvand et al. (89)	None	No	No	PFC, SMA, primary motor cortex	Brite MKIII fNIRS	Electrical task under three conditions: (1) Normal conditions. (2) Time pressure. (3) Time pressure + secondary 2-back task (respond to number sequence).
Singh et al. (67)	None	Randomized order of conditions	No	PFC	ETG-4000	Operative platform effect on prefrontal activation: Suturing on box trainer (iSim2). Two conditions: (1) Self-paced. (2) 2-min time limit per knot. Task: double throw, two single throws, 5 times each. Two sessions, second after 6 months.
Sun et al. (73)	None	Randomized pairs of teacher and student	No	PFC	ETG-4000	Task to investigate teacher-student cooperation: Tasks with cooperation and independent conditions for each dyad. Six blocks, nine trials each. Equation tasks: 5,000 ms to solve. No communication between partners during tasks.
Tang et al. (61)	None	No	No	PFC	Custom-designed, portable fNIRS system	Drone operation: area (100 m x 100 m x 100 m). Phases: (1) Empty airspace, 60 s + memorize city locations. (2) Fly to cities. (3) Fly memorized routes, 5 s stops.
Tian et al. (78)	None	No	No	dlPFC, FPC, OFC	LABNIRS	Cognitive function in Chinese coal miners during 5-min rest state: Comparing NUB and EUB; subjects still, staring at a cross for ~5 min, constant room conditions. Conducted during 10:00–14:00 to avoid work interference.
Tian et al. (43)	None	No	No	dlPFC, FPC, OFC	LABNIRS	Shift work impact on cognitive function in Chinese coal miners during 5-min rest state: Three shifts: (1) 8:00–16:00, (2) 16:00–24:00, (3) 24:00–08:00. Production (day), maintenance (night). Workday: 2 h pre-shift, 8 h shift, 2 h post-shift.
Tian et al. (85)	None	Randomized into four groups	No	dlPFC, PFC, premotor cortex	Cortivision Photon Cap	Working memory in Chinese coal miners under varying temperature and humidity conditions; four groups exposed to different combinations during 5-min resting state and short-term visual memory task.
Tyagi et al. (62)	Control group and stress group	Randomized groups	No	dlPFC, premotor cortex, SMA	NIRSport2	Pipe maintenance: turn 8 valves in order. 4 stages: (1) Familiarization: 3 operations w/wo stressors. (2) Training: 8 operations w/wo stressors. (3) Buffer: no task. (4) Evaluation: 8 operations w/wo stressors. Stressors: fires, alarms, smoke.
Verdière et al. (40)	None	Pseudorandomized assignment of landing scenarios	No	PFC, OC	NIRSport	Pilot engagement: 8 scenarios in Airbus A320 simulator: 4 manual landings, 4 automated landings.
Xu et al. (82)	None	Randomized order of conditions	No	PFC	fNIRS PioneerTM	CEM training sessions. Two trainees (provider and responder). 15 scenarios with three phases: single provider, team, debriefing. Example: “ENT Airway Fire”—manage a laser-instigated fire in patient’s mouth, determine next steps.
Zhang et al. (93)	None	Randomized order of conditions	No	PFC, MC, OC	NIR Smart fNIRS	Flying aircraft in simulator with 3 subtasks: (1) No failure. (2) Altitude and heading reference system failure. (3) Right-hand engine failure. 10 min rest between subtasks.
Zheng et al. (87)	None	Pseudorandomized splitting of students in 3 groups with 20 students each and same number of males and females	No	PFC, TC, PC	ETG-4000	Neural synchronization in teacher-student interactions: Teachers taught students (1:1) in three modes: interactive, lecturing, video. Content was the same for all groups, effects of teacher type on arithmetic test (CCSAPAT’s test).
Zheng et al. (42)	None	Pseudorandomized splitting of students in 3 groups with 20 students each and same number of males and females	No	PFC, TC, PC	ETG-4000	Task to investigate teacher-student relationship and social interaction: Teachers taught students (1:1) the same content in three modes: (1) Turn-taking mode, (2) Lecturing mode, (3) Video mode. Three different groups of individual students were used.

ATC, Air Traffic Controller; CCSAPAT’s test, Chinese Civil Servants Administrative Professional Knowledge Level Tests; CEM, crisis event management; CRA, Conflict Resolution Advisory; dlPFC, dorsolateral PreFrontal Cortex; FC, Frontal Cortex; FPC, FrontoPolar Cortex; IFG, inferior frontal gyrus; MC, moto cortex; MIST, Montreal Imaging Stress task; mPFC, medial PreFrontal Cortex; OC, occipital cortex; OFC, orbitofrontal cortex; PC, Parietal Cortex; PFC, Prefrontal Cortex; SMA, Supplementary Motor Area; RAS, Robot Assisted Surgery; TC, Temporal Cortex; UAV, Unmanned Aerial Vehicles; VPC, Ventromedial Prefrontal Cortex.

Subsequently, a third structured approach was employed to extract signal-processing components specific to fNIRS from the methods sections of the included articles. These components were categorized based on the framework established by Kohl et al. (57) and were further adapted to address the specific focus of the current research. This involved meticulous examination of fNIRS device specifications, identification of brain regions-of-interest, delineation of target regions (including channels of interest and positioning systems), preprocessing techniques, artifact control strategies [including additional measurements such as short-distance measurements, Electromyography (EMG), Electroencephalography (EEG), electrocardiogram (ECG)], selection of chromophore used for feedback (oxygenated hemoglobin – HbO, deoxygenated hemoglobin – HbR, or total hemoglobin – HbT), and the methodology employed for calculating the feedback signal. These details are comprehensively presented in Table 5.

**Table 5:** Methodological approaches for signal processing aspects and main findings.

First author (Year)	Selection of target regions (channels of interest)	Additional measures for artifact control	Chromophore used	Preprocessing	Calculation of signal information	Main findings
Abujelala et al. (60)	21 channels aligned with positions based on the international EEG 10–10 system. No information about registration were reported.	Non	HbO, HbR, HbT	Low-pass filter (3 Hz) and band-pass filter (0.5–0.016 Hz), abrupt peaks or change in the optical density signal were found and corrected using spline interpolation algorithm, and smoothed using wavelet transforms.	Average of channels / no baseline before task (not explicitly reported), 15 s sliding window¸ Min-max normalization at the participant level, connectivity attributes between cortical regions were measured by the correlation for each participant and each group HbO	Firefighting control vs. stress group under TP: ↑ HbO in stress group during TP in RDLPFC, MPFC, LDLPFC & RPM
Ahlstrom (83)	16 channels were attached to the prefrontal area. No information about a specific system or registration were reported.	Non	HbO, HbR	N/A	HbO and HbR data were averaged for each group scenario	Pilot decision-making during weather display: ↑ HbO in RPFC in during Weather avoidance flight
Aksoy et al. (71)	18 channels were attached to the prefrontal area. No information about a specific system or registration were reported.	Non	HbO	Low-pass filter (0.009 Hz), motion artifacts were corrected using a Wavelet filter (Oscillatory Dynamics Analysis (MODA) toolbox)	Average HbO across all 18 channels and over the task duration was calculated compared to 20 s baseline.	Simulated surgical environments (laparoscopic and RAS tasks): ↓HbO in PFC during RAS vs. aparoscopic
Alyan et al. (76)	37 channels aligned with positions based on the international EEG 10/10 system. Regions of interest were configured based on the MNI coordinates and registered to the MNI space.	EEG	HbO, HbR	Band-pass filter (0.02–0.5 Hz)	Change in HbO compared to 20 s baseline, all blocks combined to obtain one averaged hemodynamic response block, connectivity attributes between cortical regions measured by correlation for each period and participant in HbO	Workstation Type (SN vs. SNE) during MAST: ↑HbO in dlPFC, VPC, FPC, OFC during MAT in SE ↓HbO in dlPFC, VPC, FPC, OFC during MAT in SNE ↑ FC in PFC ↔ ↑ cognitive engagement in SE ↓ FC in PFC ↔↓ cognitive engagement in SNE
Alyan et al. (76)	52 channels aligned with positions based on the international EEG 10–20 system. No information for registration of the positions were reported.	Non	HbO	Band-pass filter (0.02–0.5 Hz), moving time-window averaging of 500 ms was used to remove high-frequency artifacts.	Changes in HbO compared to 20 s baseline, maximum *t*-value from the 52 channels	Workstation Type: SNE vs. SE: ↓ HbO in PFC during MAST at SNE vs. SE ↑HbO R-DLPFC activation during MAST at SE vs. SNE
Andreu-Perez et al. (75)	44 channels aligned with positions based on the international EEG 10–10 system. Positions were registered to MNI space.	Non	HbO, HbR	Low-pass filtered, detrended (eliminate system drift), channel rejection using ICNA software (150).	Change in HbO and HbR compared to 10 s baseline at beginning.	Skill Level in Laparoscopic Suturing: *Needle Insertion:* ↑ HbO in PFC-SMA, PFC-PMC, SMA-PMC (Novices > Trainees & Experts) *Double-Throw Knot Tying:* → HbO in PFC-PMC; ↑ FC in SMA-PMC *Single-Throw Knot Tying:* ↑ FC in PFC-PMC, PFC-SMA, SMA-M1 in Experts
Ayaz et al. (80)	16 channels attached to the participants forehead. No information about a specific system or registration were reported.	Non	HbO, HbR	Low-pass filter (0.1 Hz), no motion artifact corrections, channel rejection	Change in HbO and HbR average of all channels / no baseline before task (not explicitly reported)	Mental Workload Assessment in ATCOs: *ATC Task:* ↑ HbO in MPFC with higher Task Difficulty and Voice Communication *UAV Task:* ↓ HbO in LPFC with practice level for approach and landing tasks
Chong et al. (77)	52 channels aligned with positions based on the international EEG 10/20 system. Positions were registered to MNI space.	ECG	HbO	Low-pass filter (1.0 Hz), wavelet-based motion correction based on the hmrMotionCorrectWavelet, hemodynamic modality separation (HMS) method to separate the systemic physiological component	Pairwise correlation of wavelet coefficients (0.01 Hz to 0.2 Hz) to construct time-frequency network correlation matrices. 60s task-relevant correlations averaged to create 32 × 32 network matrices	Emotional States: Nursing Students vs. Registered Nurses: ↑ FC in RPFC during affective vs. neutral states in NS → FC changes in RN
Fan and Yang (70)	15 channels aligned with positions based on the international EEG 10/20 system. No information about registration of the positions reported.	Non	HbO	Low-pass filter (0.4 Hz), HbO data were subjected to a correlation-based transformation, CBSI applied, removing discontinuities and spike artefacts via visual inspection,	Average of channels / no baseline before task (not explicitly reported), connectivity attributes between cortical regions measured by correlation for each period and participant in HbO.	Maritime Safety in Expert vs. Novice: *Task over Time:* ↓ HbO over time in RPFC during watchkeeping *Expert vs Novice*: ↑ HbO in RPFC in Expert vs. Novice
Fan et al. (69)	15 channels aligned with positions based on the international EEG 10/20 system. No information about registration of the positions reported.	Non	HbO	Low-pass filter (0.4 Hz), HbO data were subjected to a correlation-based transformation, CBSI applied	Average of channels / no baseline before task (not explicitly reported), connectivity attributes between cortical regions measured by correlation for each period and participant in HbO.	Watchkeeping Novices vs. Experts among Seafarers: ↑ HbO in right lateral PFC for distraction group during decision-making ↑ HbO in RLPFC for experienced participants during TP ↑ FC associated with spotting target at greater distance during TP
Fan et al. (63)	20 channels aligned with positions based on the international EEG 10/20 system. Positions were registered to Brodmann’s areas.	8 short-distance channels	HbO	Band-pass filter (0.01–0.04 Hz), channel rejection, Spike removal with a threshold of 3.5, Motion correction (TDDR), Short-channel regression GLM, Correlation-based signal improvement (CBSI) and Z normalization	Average of channels and ROI / no baseline before task (not explicitly reported), connectivity attributes between cortical regions were measured by the partial correlation coefficients between channels in HbO	Watchkeeping low vs. high workload: ↑ HbO during high workload ↑ FC in PFC under conditions of high task difficulty vs. low task
Harrison et al. (74)	16 channels aligned with positions based on the international EEG 10–20 system. No information registration was reported.	Non	HbO, HbR	Low-pass filter (0.1 Hz), motion artifacts were removed through visual inspection.	Change in mean HbO and HbR compared to baseline averaging over all channels,	Conflict Resolution Advisory on ATCS’s behavior: ↑ HbO at higher AC in PFC ↑ HbO over days (Day1 vs. Day2/3) in PFC; no diffs Day2 vs. Day3
Isbilir et al. (81)	16 channels placement corresponded to the Broadman areas 9, 10, 44 and 45. No information about a specific system or registration were reported.	Non	HbT	Low-pass filter (0.1HZ), SMAR filter (145) was used to head movements, visually inspected for cases including excessive noise and motion artifacts	HbT measures were baseline-corrected with respect to the beginning of each block.	Expert vs. Novice Operators under Normal and Adverse Conditions: *Task 1 (normal condition):* → HbT Between Experts vs. Novices *Task 2 (advanced condition):* ↑ HbT in rt. PFC in Task2 vs. Task 1 ↑ HbT in rt. PFC Experts vs. Novices
Kakehashi et al. (86)	22 channels were attached to the head of the participants. No information about a specific system or registration were reported.	Non	HbO	Low-pass filter (0.1 Hz), no motion artifact corrections	Average of channels / no baseline before task (not explicitly reported)	Comfortable Lighting Locations for Office Work: Wall vs. Ceiling ↑ HbO concentration in PFC during arithmetic and copying tasks ↓ HbO in PFC under ‘wall’ illumination compared to ‘ceiling’ illumination
Kawaguchi et al. (64)	22 channels were attached to the head of the participants. Channel positions were verified by using a 3D digitizer system. Positions were registered to MNI space (NIRS-SPM software Ver. 4).	Non	HbO	Low-pass filter (0.1 Hz), no motion artifact corrections	Average concentration/ value during the flight from 1,500 ft. to 500 for each session / baseline correction (no time period)	Landing scenario for Experts (old) vs. Novices for low and high maneuvers ↑ HbO concentration in dlPFC during high vs. low in Experts ↑ HbO concentration in dlPFC during high vs. low in Novice → between Experts and Novice in HbO concentration
Le et al. (41)	4 Channels positioned on subject’s forehead. No information about a specific system or registration were reported.	Non	HbO, HbR, HbT	Band-pass filter (0.02 Hz–1 Hz), time shift (removal lost data), Kalman filtration (144).	Change in mean HbO, HbR and HbT compared to 5 s baseline	Pipe Maintenance during TP: *Stress Group:* ↓ HbO in MDLPFC, RDLPFC, & LDLPFC during Training vs. Control *Stress Group FC:* ↑ FC between LDLPFC-RDLPFC and MDLPFC-RDLPFC during Training
Li et al. (50)	16 channels aligned with the positions based on the international EEG 10/20 system. For analysis focus was set on channel 2. No information registration was reported.	Non	HbO	Low-pass filter (0.1 Hz), time shift (time window-5.0 s), artifact corrections due to DWT using reference signals.	Change in mean HbO compared to linear fitting of pre and post baselines	Mental Workload in Rail Transit Drivers: ↑ HbO levels during DT indicating increased PFC activity due to mental workload
Li et al. (92)	22 Channels aligned with positions of EEG 10–20 system. Channel positions were verified by using a 3D digitizer system.	Non	HbO	Band-pass filter (0.01 Hz–0.2 Hz), SMAR at 0.5 Hz, time-frequency waveform analysis	Connectivity attributes between cortical regions were measured by the correlation between blocks in HbO	Optimized vs. Descriptive Map in ACT Task: ↑ FC between LPFC, RPFC & ROL
Li et al. (90)	48 channels attached to the PFC No information about a specific system or registration were reported.	Non	HbO	N/A	Average concentration HbO compared to 2 min baseline	→ HbO changes between Imtervention and Control → HbO changes in post Task 8 and 9 in Intervention or Control
Liu et al. (72)	22 channels aligned with positions of EEG 10–20 system. Channel positions were verified by using a 3D digitizer system (NirMap). Positions were registered to MNI space (NIRS-SPM software Ver. 4).	Non	HbO	Channel rejection, motion artifacts were corrected using a 5 s sliding-window motion artifact rejection (SMAR)	Average of channels and ROI compared to 10 s baseline, GLMs estimated β-values	Mental health status of employees in high-tech companies: ↑ HbO in right DLPFC and bilateral Broca’s area in R&D group compared to sales group during VTF ↓ SCL-90 score in in R&D group compared to sales group
Menda et al. (79)	16 channels attached to the forehead. No information about a specific system or registration were reported.	Non	HbO, HbR, HbT	Low-pass filter (0.2 Hz), no motion artifact corrections	Data were averaged over 100 s before and after each trial for each subject. HbT concentration changes were calculated for pre-and post-blocks and normalized using z-score calculation for each pair independently.	UAV Operator’s Cognitive Workload: Onboard vs. Chase View: ↑ HbO in PFC during Onboard View vs. Chase View ↑ HbO in RPFC higher than LPFC under all conditions
Midha et al. (51)	8 channels attached to the PFC. No information about a specific system or registration were reported.	Non	HbO	Band-pass filter (0.01 Hz–0.5 Hz), motion artifacts were corrected using a Wavelet filter, physiological noise was reduced using a PCA	Change in HbO compared to 10 s baseline, contrast between the t-values estimated for different conditions	Reading and Writing Task Difficulty with Interruptions: ↑ HbO in LPFC during hard reading condition → No significant differences in brain activity between writing conditions
Modi et al. (68)	24 channels aligned with positions based on the international EEG 10–20 system. No information about registration of the positions reported.	Non	HbO, HbR	Low-pass filter (0.5 Hz), channel rejection was based on amplitude thresholding and a signal-to-noise ratio of 1, channel-wise motion detection and spline correction were performed via visual inspection	Change in mean HbO and HbR in each channel and first, middle and last minute of task condition compared to baseline >1 min,	Resident Operative Experience: Senior vs. Junior Residents under Temporal Pressure: *Laparoscopic Suturing:* ↓ HbO in PFC in junior residents under TP *Senior Residents:* ↑/→ HbO in PFC under TP compared to SP
Modi et al. (65)	24 Channel aligned with positions of the international 10–5 system. Positions assessed via individual digitizer measurements.	Non	HbO	Low-pass filter (0.5 Hz), data rejection rate = 1%; Channel data was detrended to correct for baseline fluctuations and averaged across blocks	Change in mean HbO and HbR compared to 10 s baseline	Surgical Residents: Q1 vs. Q4: Q1: ↑ HbO in bilateral VLPFC & RDLPFC during SP & TP Q4: ↓ HbO in PFC during SP & TP GLM Findings: Q1: ↑ then ↓ HbO concentration Q4: ↓ then → HbO concentration
Mora et al. (66)	18 channels aligned with positions based on the international EEG 10/20 system. No information about registration of the positions reported.	Non	HbO, HbR	Band-pass (0.01 Hz–0.5 Hz), motion-related artifacts corrected using wavelet-based procedure (143), channel rejection for given participants.	Used onset and duration of each condition as predictors in GLMs Estimated β coefficients for each condition and within each channel β coefficients indicate direction and intensity of cortical activity change	Pesticide Exposure and Cortical Brain Activation among Farmworkers: ↓ HbO in LDLPFC during STAT associated with higher urinary TCPy concentrations ↓ HbO in bilateral PFC during working memory tasks associated with higher 3-PBA and DCCA concentrations
Naik et al. (91)	44 channels aligned with positions based on the international EEG 10/20 system.	EMG/ECG	HbO	Fourth order low-pass filter, data normalisation	Change in mean HbO	Simulated laparoscopic performance during auditory N-back task with varying difficulty: ↑ HbO correlates with increased task difficulty Multimodal measurement methodology with objective procedures; classification accuracy of 97% for CWL levels based on confusion matrix and cross-validation results.
Palzes et al. (45)	18 channels aligned with positions based on the international EEG 10/20 system. No information about registration of the positions reported.	Non	HbO, HbR	Band-pass filter (0.01–0.5 Hz), corrected for motion-related artifacts using a wavelet-based correction procedure (143)	GLM analysis, contrast between the t-values estimated for these conditions within the GLM procedure for HbO and HbR, functional localization procedure by selecting the channel with the greatest contrast value for use in group-level analyses	Min Exposure and Brain Activity in Farmworkers: ↑ HbO correlated with increased task accuracy (not significant after FDR correction) in dlPFC → No association between Mn concentrations and task accuracy
Pooladvand and Hasanzadeh (88)	20 channels attached to the head above the Brodmann areas. No information about a specific system or registration were reported.	Non	HbO	Low-pass filter (0.5 Hz), no motion artifact corrections, channel rejection	GLM analysis, average of channels/ no baseline before task (not explicitly reported), IME windows of 1 s, 3 s, 5 s, and 10 s were tested over the normalized Hbo data	Decision-making in stressful situations in Emergency Response Training: ↑ HbO in RPFC & LPFC & SMA when comparing Condition, I and III ↑ HbO in RPFC vs. LPFC under all conditions
Pooladvand et al. (89)	20 channels attached to the head above the Brodmann areas. No information about a specific system or registration were reported.	Non	HbO	Low-pass filter (0.5 Hz), no motion artifact corrections, channel rejection	GLM analysis, average, maximum, standard deviation, skewness, and kurtosis values of Hbo of channels and concentration no baseline before task (not explicitly reported), Time windows applied of 1 s, 3 s, 5 s, and 10 s were tested over the normalized Hbo data	Cognitive workload under time pressure in construction simulation training: 3-s window that obtained an accuracy of 89% for classifying cognitive loads The average and maximum Hbo values were the most accurate ↑ HbO in RPFC & LPFC & SMA when comparing condition hard vs. low load
Singh et al. (67)	24 channels aligned with positions based on the international EEG 10–20 system. No information about registration of the positions reported.	Non	HbO, HbR	Low-pass filter (0.5 Hz), channel rejection was based on amplitude thresholding and a signal-to-noise ratio of >2, motion artefacts corrected using spline interpolation via visual inspection	Change in mean HbO and HbR in each channel and each condition compared to 10 s baseline	Robotic Surgery vs. Conventional Laparoscopy under TP: *Laparoscopic Suturing:* ↓ HbO responses in TP compared to SP in bilateral VLPFC and DLPFC (↓) *Robotic Suturing:* ↑ HbO responses in TP compared to SP in bilateral VLPFC (↑)
Sun et al. (73)	22 Channel aligned with positions of the EEG 10–20 system with lowest probe at the frontal pole midline point. Positions were assessed with a virtual registration method.	Non	HbO	No filtering or detrending in pre-processing. No artifact corrections due to WTC normalization	Hyperscanning; WTC toolbox calculate two time-series by measuring cross correlations as a function of frequency and time; cross-correlation between rest and task state period; calculated the task-related coherence between subject groups (ET and NT) across all channels.	Effects of Teacher Type on Cooperative Performance: ↑ BS in ET-S dyads: significant IBS at left DLPFC during cooperation No sig. IBS in other conditions ↑ BS ↔ PTA & accuracy rates in ET-S dyads
Tang et al. (61)	8 channels divided into 2 pads positioned on subject’s forehead. No information about a specific system or registration were reported.	Non	HbO, HbR	Low-pass filter (0.3 Hz); Data extracted 5 s before onset to task completion. No artifact corrections reported	Change in mean HbO and HbR compared to 5 s baseline	Flight Performance: Trainee vs. Experienced Pilots: ↓ HbO & HbT in RPFC & LPFC from Day 1 to Day 5 & ↓ task duration over days Day 5: ↓ HbO vs. Experienced Pilots & ↑ performance vs. Experienced Pilots Flight performance ↔ HbO (↑ HbO with ↑ task duration); ↓ HbR over days
Tian et al. (78)	22 channels attached to the prefrontal area using the detector 7 for accuracy of positioning. Channel positions were verified by using a 3D digitizer system.	Reference channel	HbO	Band-pass filter (0.02–0.08 Hz), artifact corrections due to DWT	average of channels / 5 baseline before task, maximum t-value from the 22 channel, connectivity attributes between cortical regions were measured by the correlation for each group in HbO	Unsafe Behaviors: EUB vs. NUB: ↑ FC in PFC (FPA, dlPFC) in EUB vs. NUB during rest state; ↑ More intensive FC in dlPFC and FPA in EUB
Tian et al. (43)	22 channels attached to the prefrontal area using the detector 7 for accuracy of positioning. Channel positions were verified by using a 3D digitizer system.	Reference channel	HbO	Band-pass filter (0.02–0.1 Hz), artifact corrections due to Wavelab850 toolbox	Average of channels / 5 baseline before task, maximum *t*-value from the 22 channel, connectivity attributes between cortical regions were measured by the correlation for each group before and after the shift in HbO	Shift work in coal miners at rest before and after the shift: ↓ FC in morning & afternoon shifts Morning: diffs in dlPFC, dlPFC-FPC, dlPFC-OFC, OFC Afternoon: diffs in dlPFC, dlPFC-FPC, FPC, FPC-OFC, dlPFC-OFC ↑ FC in night shifts
Tian et al. (85)	27 Channel aligned with positions of the international 10–5 system No information for registration of the positions were reported.	Six short-distance channels	HbO	Band-pass filter (0.01–0.1 Hz), Motion correction (TDDR),	GLM analysis using average and maximum HbO values; no baseline reported prior to task. Short-channel regression applied. GLM-derived β-values used as indicators of brain activity per channel.	Effects of temperature and humidity on working memory in coal mine workers: ↑ HbO in dlPFC under high temperature and humidity ↓ Reaction times and impaired working memory performance
Tyagi et al. (62)	21 channels aligned with positions based on the international EEG 10–10 system. Brodmann areas, corresponding anatomical locations were reported.	Non	HbO	Band-pass filter (0.01 Hz–0.5 Hz), motion artifacts identified in each channel by sharp fluctuations along with 0.75 s before and after the segment	Channels were split into six regions of interest. Peak HbO values were obtained and averaged for each region. Trials were averaged over phases. Connectivity attributes between cortical regions were measured by correlation for each participant and group in HbO.	Emergency Response in firefighting Under Stress condition: ↓ HbO in MDLPFC, RDLPFC, and LDLPFC compared to CG in Stress ↑ FC between LDLPFC-RDLPFC and MDLPFC-RDLPFC in Stress Group vs. CG
Verdière et al. (40)	Two NIRSport in tandem mode resulting in 42 channels. No information about a specific system or registration were reported.	Non	HbO, HbR	Band-pass filter (0.01 Hz–0.5 Hz), wavelet interpolation method for the artifact correction (148)	Average of channels / no baseline before task (not explicitly reported) connectivity attributes between cortical regions were measured by the correlation for each participant and each group in HbO and HbR	Pilot Engagement: Connectivity vs. Classical Features: ↑ Conn. Features > Class. Features in classification accuracy in PFC → Chromophore Type Not Significant ↑ Feature Type Affects Classification Performance ↑ Conn. Features > Class. Features
Xu et al. (82)	1 channel attached on the right side of the anterior prefrontal cortex with position based on the international EEG 10–20 system. No information about registration of the positions reported.	Non	HbO, HbR	Band-pass (0.01 Hz–0.1 Hz), wavelet-based motion artifact removal procedure to all HbO and HbR time series	Change in mean HbO and HbR in each channel and condition compared to baseline >1 min.	Examine Team Experience During Conflict Task: ↑ HbO in PFC in team/debriefing phases vs. single provider phase in low difficulty scenarios → HbO/HbR correlation between observer-rated workload, self-reported workload, or mood measures ↑ FC in PFC in high difficulty scenarios vs. in low difficulty scenarios
Zhang et al. (93)	38 channels aligned with positions based on the international EEG 10–20 system. No information about registration of the positions reported.	Non	HbO	Band-pass (0.01 Hz–0.2 Hz), artifact removal by spline interpolation	Change in HbO average of all channels and each subtask period / no baseline before task (not explicitly reported)	Pilots’ Mental Workload Under Emergency Flight Conditions: ↑ HbO in PFC and MC from low and middle workload ↓ HbO in PFC in high workload
Zheng et al. (87)	20 channels aligned with positions based on the international EEG 10–20 system. Positions were registered to MNI space.	Non	HbO	No filtering or detrending in pre-processing. No artifact corrections due to WTC normalization (147, 149), discarded unstable data from session edges (10s).	Hyperscanning; WTC toolbox calculate two time-series by measuring cross correlations as a function of frequency and time; cross-correlation between rest and task state period; calculated the task-related coherence between each pair of participants across all channels.	Teaching Style Impact on INS and Teaching Outcomes: *Teaching Outcome ↔ INS at aSTC-TPJ:* Significant positive correlation between teaching outcome and INS between teacher’s aSTC and student’s TPJ; *Teaching Style ↔ INS at TPJ-TPJ:* Significant effect of teaching style on INS increase at right TPJ-TPJ Video style had lower INS increase than other styles
Zheng et al. (42)	10 Channels aligned with positions of the EEG 10–20 system. Positions were registered to MNI space. Channel positions were verified by using a 3D digitizer system.	Non	HbO	No filtering or detrending in pre-processing. No artifact corrections due to WTC normalization (147, 149), discarded unstable data from session edges (10s).	Hyperscanning; WTC toolbox calculate two time-series by measuring cross correlations as a function of frequency and time; cross-correlation between rest and task state period; calculated the task-related coherence between each pair of participants across all channels.	Interaction: Teachers vs. Students: ↑ BS in right SMC post-interaction in turn-taking mode only ↑ BS more closely associated with affiliative attachment than with social interaction

3-PBA, 3-Phenoxybenzoic Acid; ↑: Increase; →: No Significant Change; ↓: Decrease; &: And; ↔: Association or Correlation; AC: Aircraft Count; aSTC: Anterior Superior Temporal Cortex; ATC: Air Traffic Control; ATCO: Air Traffic Control Officer; BA10: Brodmann Area 10; BS: Brain Synchronization; CBSI: Correlation-Based Signal Improvement; CEM: Crisis Event Management; CG: Control Group; CRA: Conflict Resolution Advisory; DCCA: cis-DCCA and trans-DCCA; DLPFC: Dorsolateral Prefrontal Cortex; DT: Determination Test; DWT: Discrete Wavelet Transformation; EUB: Engaged in Unsafe Behaviors; ET-S: Expert Teacher-Student; FC: Functional Connectivity; FDR: False Discovery Rate; FPA: Frontal Pole Area; FPC: Frontopolar Cortex; GLM: General Linear Model; HbO: Oxyhemoglobin; HbR: Deoxyhemoglobin; HbT: Total Hemoglobin; HRV: Heart Rate Variability; IBS: Inter-Brain Synchronization; ICNA: Imperial College Neuroimage Analysis; INS: Interpersonal Neural Synchronization; LDLPFC: Left Dorsolateral Prefrontal Cortex; LPFC: Left Prefrontal Cortex; M1: Primary Motor Cortex; MAP: Mean Aircraft Performance; MC: Motor Cortex; MDLPFC: Middle Dorsolateral Prefrontal Cortex; ML: Machine Learning; MNI: Montreal Neurological Institute; Mn: Manganese; MPFC: Medial Prefrontal Cortex; NS: Nursing Students; NUB: Not Engaged in Unsafe Behaviors; OFC: Orbitofrontal Cortex; PCA: Principal Component Analysis; PFC: Prefrontal Cortex; PMC: Premotor Cortex; PTA: Perspective-Taking Ability; Q1: Top Quartile; Q4: Bottom Quartile; RAS: Robot Assisted Surgery; R&D: Research and Development; R&D: research and development; RDLPFC: Right Dorsolateral Prefrontal Cortex; RLPFC: Right Lateral Prefrontal Cortex; RN: Registered Nurses; ROI: Region of Interest; ROL: Right Occipital Lobe; RPM: Right Premotor Cortex; RPFC: Right Prefrontal Cortex; SE: Ergonomic Workstation; SI: Social Interaction; SMA: Supplementary Motor Area; SMAR: Sliding-Window Motion Artifact Rejection; SMC: Sensorimotor Cortex; SNE: Non-Ergonomic Workstation; SP: Self-Paced; TCPy: 3,5,6-Trichloro-2-pyridinol; TP: Time Pressure; TPJ: Temporoparietal Junction; UAV: Unmanned Aerial Vehicle; VFT: verbal fluency test; VLPFC: Ventrolateral Prefrontal Cortex; WTC: Wavelet Transform Coherence.

Both extraction and assessment of all method-specific information were conducted by RG, with validation of accuracy by OM.

### 2.6 Study quality

To evaluate the methodological quality of the included studies, we used the checklist from the Joanna Briggs Institute (JBI) Critical Appraisal Tool for quasi-experimental studies (58). Although the PICOS framework was considered, the JBI tool was deemed most appropriate due to its stringent criteria tailored to quasi-experimental designs, which are commonly used in systematic reviews when randomized controlled trials (RCTs) are unavailable or scarce. The revised JBI tool integrates recent advancements in bias assessment, making it an optimal choice for this review. Despite recognizing some limitations, we believe this approach achieves the best balance between thorough assessment and methodological rigor. Two of the authors (RG, OM) independently assessed the studies based on the nine criteria of the checklist. These criteria encompass clarity of cause and effect (temporal relationship between variables), similar participants, similar treatment in compared groups, the existence of a control group/condition, multiple measurement points of the outcome, completion of follow-up, similar outcome measurements in compared groups, reliability of outcome measurements and appropriate statistical methods.

Points were allocated to each study based on the number of criteria fulfilled. While we set a criterion of 7 or more ‘yes’ responses to assess the methodological quality, no studies were excluded from the synthesis based on a lower score. This approach aligns with the general recommendation from JBI to include all eligible studies in the synthesis, ensuring a comprehensive analysis of the available evidence (58, 59). Disagreements between the reviewers were resolved either by reaching a consensus or consulting a third reviewer (AS). For further details about rating criteria, see Supplementary Table S1.

## 3 Results

### 3.1 Identification of studies

**Figure 1:**
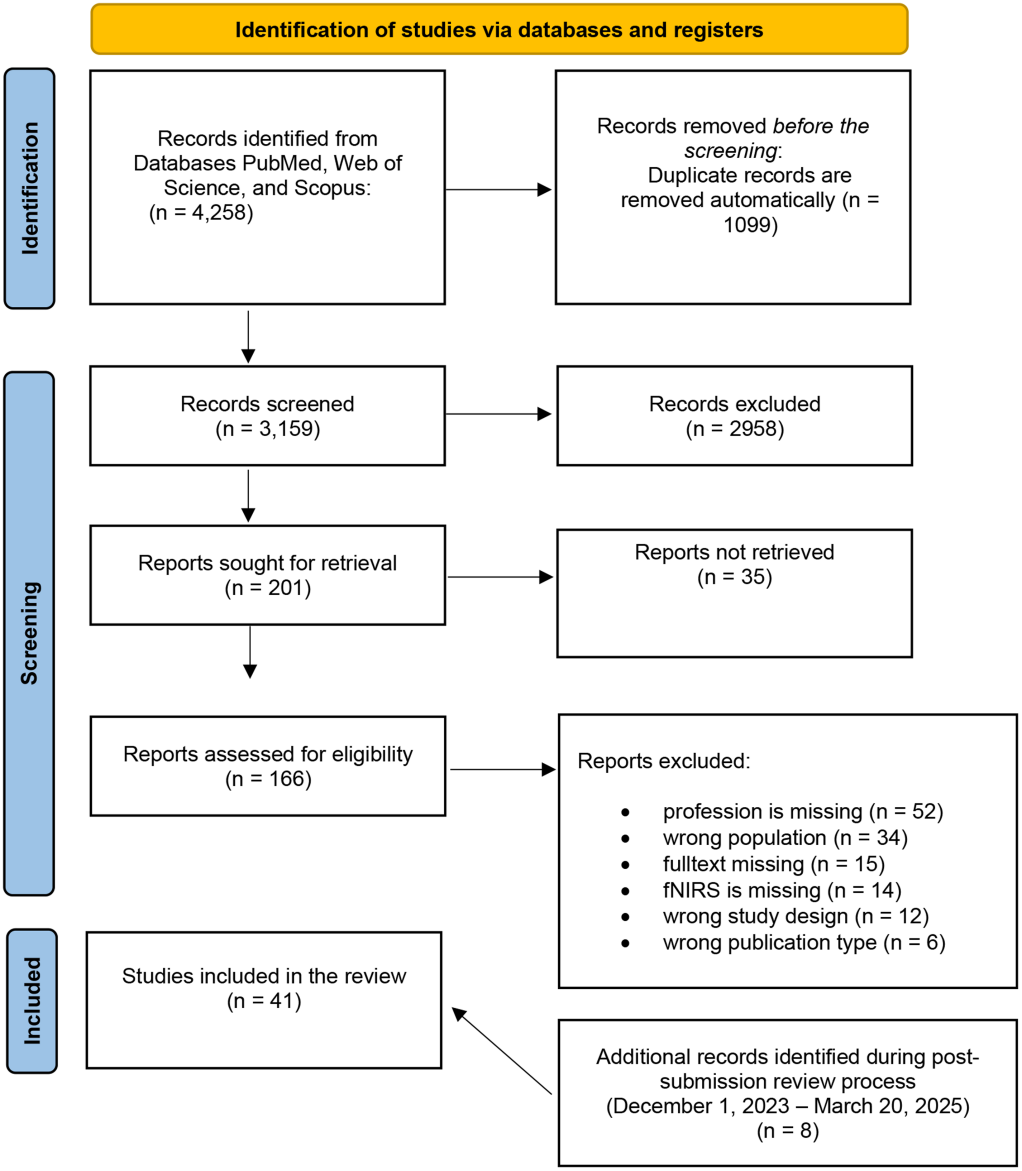
PRISMA 2020 flow diagram for new systematic reviews which included searches of databases, and registers.

The systematic search resulted in a total of 4,258 articles. After the automated removal of duplicates from the record list using the data management software *Rayyan* and the subsequent title and abstract screening, a total of 201 articles were identified as potentially relevant, and eligibility was assessed. In the conclusive stage of the full-text screening, 33 articles were deemed pertinent for subsequent data extraction. To ensure that the review reflected the most recent state of research, an additional screening step was conducted, resulting in the identification and inclusion of 8 further eligible studies. These were identified after the initial submission, during the peer-review process, and were fully integrated into the synthesis. In total, 41 studies were included in the final review. A schematic overview of the study selection process is presented in Figure 1.

### 3.2 Study characteristics

Table 3 summarizes the characteristics of the 41 included studies. Sample sizes ranged from 5 participants (41, 60) to 154 participants (61). 15 studies included exclusively (62–64) or predominantly male participants (>80%) (40, 41, 45, 50, 65–72). Only one study (73) focused primarily on female participants, while four reported a balanced gender distribution. 15 studies did not report gender information (43, 60, 61, 74–85). The participants’ age range varied widely across studies. For example, trainee pilots had an average age of 21.1 (± 2.1) (61) and experienced seafarers averaging *M* = 44.6 years (± 15.5) (69, 70).

### 3.3 Occupation and professional experience in years

The studies covered a variety of occupations and work environments. Sedentary work environments were represented in studies on office work (51, 72, 76, 84, 86), and teachers (42, 73, 87). Physically active occupations included firefighters (60, 62), miners (43, 78, 85), agricultural workers (45, 66), military personnel (81), seafarer (63, 69, 70), and electrical line workers (88, 89). Medical professions were represented by surgeons (65, 67, 68, 71, 75, 90, 91), anesthesiologists (82), and nurses (77). The aviation and transportation sector was represented by drivers (41) air traffic controllers (50, 74, 80, 92), pilots (40, 64, 80, 83, 93), drone pilots (61, 79), and subway drivers (50).

Participants’ professional experience varied widely, ranging from 1 year (68, 71, 82, 88–90) to over 30 years (45, 80). Ten studies compared different levels of professional experience, for example between experts and novices, about job-specific tasks (61, 64, 68–70, 73, 75, 81–83). In 13 studies, the participants’ professional experience was not specified (40, 41, 51, 63, 67, 72, 74, 76, 79, 81, 84, 86, 92).

### 3.4 Types of workloads

The focus of the investigated occupational workload was primarily on mental workload (7, 41, 50, 51, 60–65, 67, 68, 70, 71, 76, 77, 80, 83, 89, 93) and cognitive workload (40, 43, 66, 71, 74, 75, 79, 81, 82, 86, 91). Mental workload refers to the cognitive demands of processing, storing, and managing information during task performance, influenced by task complexity, environment, and individual capabilities (4–6). Cognitive workload, based on Cognitive Load Theory (2, 3) describes the mental effort needed to process and integrate information, shaped by task complexity, instructional design, and cognitive resources. Although we distinguished between mental and cognitive workload based on each publication’s terminology, both terms refer to the cognitive demands and effort exerted during occupational tasks. For our purposes, we treated them as functionally equivalent under the broader concept of occupational workload. Among the studies included, six studies also investigated temporary stress (65, 68), or time pressure (60, 62, 67, 90) in addition to mental or cognitive workload. In total, 29 studies examined the effects of occupational activities on cognitive and mental workload, occasionally under additional stress while performing or learning job-specific tasks (67, 72).

Five studies investigated decision-making in job-specific contexts (63, 69, 88, 89, 92). Another three studies focused on teacher-student interactions, including cooperative performance, quality of interaction, and affective commitment (42, 73, 87). Two studies analyzed the effects of pesticide exposure on the cognitive functioning of agricultural workers (45, 66). One study investigated mental fatigue and functional brain connectivity in miners (78).

### 3.5 Design aspects

#### 3.5.1 Methodological quality (JBI critical appraisal tool)

Supplementary Table S1 summarizes the assessment results for each study. On average, the studies met 3.6 (SD = 2.09) of the 9 essential quality criteria, thus receiving a ‘yes’ rating. Notably, only seven studies were deemed to employ appropriate statistical methodologies. Many studies were considered deficient in statistical rigor due to inadequate justification of their sampling plans or failure to classify their research as pilot, feasibility, or concept studies. Additionally, the lack of control groups further contributed to the methodological shortcomings of these studies, significantly diminishing their scientific robustness. Importantly, question 9, which concerns statistical conclusion validity, was not included in the overall risk of bias assessment, aligning with the guidance of the JBI tool to focus solely on internal validity (59).

#### 3.5.2 Randomization

Of the 41 included studies, four randomized the assignment of participants to different groups (62, 73, 85, 90). Eight studies randomized the sequence of conditions within the utilized conflict tasks (63, 65, 67, 68, 71, 82, 83, 93), such as the randomization of condition employed in various flight scenarios (69, 82, 93). Additionally, five other studies used pseudo-randomization for group assignment (42, 87) the order of conditions (40, 80), or the order of participants (74). One study used quasi-randomized group for distraction task (69, 70).

#### 3.5.3 Control conditions

Only six of the 41 studies included a control group in their study design. Two studies divided firefighters into stress and control groups to compare prefrontal cortex brain activity during occupational workload under time pressure in a between-subjects design (60, 62). Additionally, two other studies compared the effects of pesticides in agriculture on working memory performance and prefrontal cortex activity between organic and conventional farmers in a between-subjects design (45, 66). Menda et al. (79) divided unmanned aerial vehicle (UAV) pilots into two groups: Group 1 followed tasks from a chase view, while Group 2, serving as the control group, used the onboard camera interface to investigate the use of fNIRS for monitoring cognitive workload and situational awareness of UAV operators during simulated missions in a between-subjects design. Li et al. (90) examined whether a two-week mindfulness training program could enhance performance under time pressure in a simulated knee arthroscopy task. The intervention group received structured mindfulness training, while the control group underwent no additional training.

#### 3.5.4 Follow-up measures

Six studies assessed the effects of occupational workload with follow-up periods measurements ranging from several days to multiple weeks. Two studies focused on air traffic controllers: one examined the stability of cognitive performance across repeated sessions (92), while the other investigated the influence of conflict resolution advisories on workload during a multi-day training course (74). Other follow-up designs included the assessment of sustained functional connectivity in nurses (77), the consistency of neurophysiological responses in urban rail drivers (50), and the long-term effects of pesticide exposure in farmworkers (66). Additionally, one study conducted follow-up measurements to evaluate the effects of a two-week mindfulness intervention on surgical performance under time pressure in orthopedic training (90).

### 3.6 Signal processing aspects

#### 3.6.1 Devices

Fifteen different devices were used across the studies: ETG-4000, OT-R40, NIRSport/NIRSport 2, NIR Smart fNIRS, NIRSIT, ASTEM’s fNIRS, LABNIRS, fNIRS PioneerTM, fNIRS Imager 1,002, Brite MKIII, Artinis Brite24, BS-3000, fNIR Devices LLC, NIRSport 88, LIGHTNIRS, WOT-100 System, and Octamon. Additionally, one study employed a custom-developed portable fNIRS system (61), and another study did not specify the device manufacturer (79), as shown in Table 5. The frequency of use of certain devices does not imply qualitative superiority. The primary differences between the devices include the wavelengths used and the number of wavelengths supported by the systems. Some devices use two wavelengths (e.g., ETG-4000, NIRSport/NIRSport 2, Brite MKIII, Brite24), while others support three wavelengths (e.g., OT-R40, LABNIRS). Each device utilizes different wavelengths within the range of 690 to 860 nm, and they exhibit varying degrees of accuracy (e.g., dynamic range or sensitivity), depending on the hardware and its quality.

#### 3.6.2 Target brain regions: selection of channels of interest

The majority of studies focused on investigating cortical activity in the prefrontal cortex (PFC), particularly the dorsolateral prefrontal cortex (dlPFC), the frontopolar cortex (FPC), and the orbitofrontal cortex (OFC). The motor cortex (MC), including the premotor cortex, the supplementary motor area (SMA), and the primary motor cortex, was also a frequently studied area. Cortical activities in the temporal cortex (TC), parietal cortex (PC), and occipital cortex (OC) were only analyzed in a few studies. One study additionally focused on Broca’s area in the left hemisphere (66).

To identify specific brain regions, the studies employed various methods for optode placement on participants’ heads. All studies relied on *a priori* knowledge of brain region locations. Among the 41 studies, 20 used the EEG 10–20 system (94) as a reference for optode placement (42, 45, 50, 63, 65–70, 73–77, 82, 87, 91–93), while six used the EEG 10–10 system (60, 62, 75, 84) or the 5–10 system (65, 85). Additionally, seven studies utilized the MNI space registration methods outlined by Okamoto et al. (95), Singh et al. (96) and Tsuzuki et al. (42, 64, 72, 75, 77, 84, 87, 97). Additionally, six studies performed 3D digitizer measurements to verify optode positions (42, 43, 64, 72, 78, 92). Conversely, 13 studies provided no specific information on additional systems or optode registration methods (40, 41, 51, 61, 70, 71, 79–81, 83, 86, 88, 90). The number of channels used varied between 1 and 54.

#### 3.6.3 Chromophores and visualization of cortical activity

To visualize cortical activity using fNIRS, 26 of the 41 studies used HbO as the preferred chromophore (42, 43, 50, 51, 60, 62–65, 69–73, 76–78, 85–93). Eleven studies used both HbO and HbR (40, 45, 50, 66–68, 74, 75, 80, 82–84). Three studies used all three chromophores: HbO, HbR, and HbT (10, 60, 79). Only one study used HbT to derive cortical activity (81) (see Table 5).

#### 3.6.4 Signal pre-processing and artifact control

The fNIRS signal is subject to various sources of noise, particularly physiological noise such as low-frequency blood pressure oscillations (Mayer waves) (98, 99), vasomotor interference (100), and movement artifacts (e.g., head movements) (101). These interferences can overlap with the task frequency. Various preprocessing methods were used to reduce the noise. In 22 studies, the signal was filtered using low-pass filters (50, 60, 61, 64, 65, 67–71, 74–77, 79–81, 84, 86, 88, 89, 91). 17 studies used band-pass filters, which included both low-pass and high-pass filters (40, 41, 43, 45, 51, 60, 62, 63, 66, 73, 76, 78, 82, 84, 88, 92, 93). Six studies did not report or use a filtering method (42, 73, 76, 83, 87, 90).

Four studies that applied artifact control used either reference channels (43, 78) or ECG measures for *post-hoc* artifact control to detect changes in heart rate (77, 91). Two study used *post-hoc* EEG measurement for artifact control and compared neuronal activity in synchronization measurements of brain connectivity with EEG and fNIRS (84, 91). 15 of 41 studies did not use or report any explicit artifact control on top of the filtering methods (42, 61, 64, 69, 72, 73, 79, 80, 83, 84, 86–90). Three studies used a sliding-window motion artifact rejection (SMAR) procedure which rejected motion-affected periods in the fNIRS signal (72, 81, 92). 14 studies used wavelet filters for real-time spike artifact removal (40, 43, 45, 50, 51, 60, 62, 63, 66, 71, 76–78, 82). Three studies removed motion artifacts through visual inspection (67, 70, 74). Eight studies applied a channel rejection based on amplitude thresholding and a signal-to-noise ratio from 1 to >2 (63, 67, 68, 72, 80) or applied channel rejection without any further information (75, 80, 88). Two study implemented short-distance channels for artifact control and applied short-channel regression within a general linear model (GLM) framework (63, 85).

#### 3.6.5 Data processing and calculation of workload information

In almost all of the studies reviewed, baseline correction and averaging (e.g., across channels, trials, and/or distinct time periods) were conducted. The average cortical activity measurements for the chromophores used (HbO, HbR, and HbT) during the task block were generally used and compared either to a pre-recorded baseline (41, 43, 51, 61, 64, 65, 67, 68, 71, 72, 75, 76, 78, 79, 82, 84), the fNIRS-system baseline (42, 73, 81, 87), or a GLM baseline (45, 66, 88, 89). The length of the baselines varied as follows: Four studies used a baseline of ≤5 s after the trial onset (41, 43, 61, 78), four studies used a baseline of 10 s after the trial onset (51, 65, 72, 75), and seven studies used a baseline of ≥10 s after the trial onset (68, 71, 76, 79, 82, 84, 90). 13 studies did not provide length specifications for the baseline comparison (40, 42, 50, 60, 63, 64, 69, 70, 74, 80, 81, 86, 93). In other studies, the peak value of cortical activity proxies over a specific time period was used for statistical analysis (76, 78).

### 3.7 Cortical hemodynamics during occupational workload

Most studies assessed cortical activity, (HbO and HbT), during occupational workload. A total of 16 studies integrated standardized neurobehavioral tasks alongside cortical measurements. Of these, seven employed the n-back task to assess working memory or cognitive workload (41, 50, 80, 88, 89, 91, 92), or a short-term visual memory task (85). Three studies used the Montreal Imaging Stress Task (MIST) to induce cognitive stress (76, 84, 86). Two applied a modified Sternberg letter-retrieval task to evaluate short-term memory (45, 66). One study combined a Go/No-Go task with the Wisconsin Card Sorting Test to assess executive functions (66). Only two studies investigated the effects of occupational workload on cortical activity during subsequent rest phases (43, 78).

Twenty three of the 41 studies reported increased HbO or HbT levels in the PFC during occupational workload. These studies spanned various professional sectors. Five studies focused on office work settings (51, 72, 76, 84, 86). Seven studies involved physically active occupations, such as maritime navigation and manual labor (45, 63, 69, 70, 81, 88, 89). Four studies examined medical professionals performing tasks such as surgery (65, 67, 82, 91). Additionally, five studies investigated individuals in the aviation and transportation sectors (64, 74, 80, 92, 93). Four studies also reported increased activity in the SMA, motor cortex, and premotor cortex during occupational tasks (76, 84, 88, 93). Functional connectivity (FC), reflecting interactions between brain regions, was analyzed in five studies. Increased FC between the PFC and FPA was observed across professions, including seafarers (63, 69), air traffic controllers (92), drivers (41), firefighters (62), and student nurses (77). Two studies reported increased FC between the FPA and dlPFC during rest in miners (43, 78). Additionally, two studies found increased brain synchronization during teacher-student interactions in both interactive and lecture settings (42, 87).

Conversely, several studies reported significant decreases in HbO concentrations during occupational workload. Participants using an ergonomic workstation exhibited decreased HbO levels in the medial, right, and left dlPFC during the MIST (86). HbO decreased over time in the right PFC during maritime watchkeeping tasks (70). Under additional time pressure, decreased HbO in the dlPFC was observed in firefighters (62), drivers (41), and junior surgical residents (65, 68, 71). High workload during flight simulator tasks led to decreases in HbO in the PFC and motor cortex (93). Additionally, morning and afternoon shifts reduced FC in the dlPFC during rest among coal miners (43).

Ten studies examined cortical activity differences between experts and novices during occupational workload. Experts generally exhibited increased HbO in the PFC compared to novices. This was evident in seafarers during decision-making tasks involving spotting distant targets (69, 70), surgeons performing laparoscopic suturing under time pressure (65, 68), and operators of military land platforms during advanced tasks (81). Experts also showed increased FC between the PFC and motor areas during laparoscopic suturing under time pressure (75). Teacher-student cooperative tasks revealed increased brain synchronization in the left dlPFC in expert dyads compared to novice dyads (42, 87). Conversely, experienced drone pilots (61) and surgeons (65) exhibited decreased HbO levels in the PFC over the task duration compared to novices.

## 4 Discussion

In this systematic review, we analyzed 41 studies that utilized fNIRS to measure cortical activity in healthy adult workers during occupational tasks. We extracted methodological details such as study characteristics, design aspects, and signal processing methods. Additionally, we collected information on occupational workload and the main findings related to cortical hemodynamics during work-related tasks. The primary goal was to determine how fNIRS is employed to investigate occupational workload.

The results demonstrate that fNIRS is effectively used to assess workload in various professions. Different occupational tasks are associated with specific changes in cortical hemodynamics, particularly in the PFC. In safety-critical and cognitively demanding fields such as aviation, maritime navigation, and medicine, a significant increase in HbO and HbT concentrations within the PFC was frequently observed during occupational workload (45, 63–65, 67, 69, 70, 74, 80–82, 88, 89, 91–93). The PFC is crucial for executive functions such as working memory, attention regulation, and decision-making (27, 102). The observed hemodynamic responses suggest heightened neural activation due to increased cognitive demands and complex decision-making inherent in these professions. This finding aligns with cognitive load theory and mental workload models, which propose that higher task demands require more cognitive resources, leading to increased neural activity in executive brain regions (4, 6). For example, experienced seafarers showed increased PFC activation during navigation-related decision-making tasks, which correlated with better task performance. This suggests efficient use of executive functions to manage complex operational environments (63, 70). These results are consistent with neuroimaging studies associating increased PFC activation with higher cognitive load and engagement of executive control networks (103–106). However, these cortical activations should not be considered as linear indicators of task difficulty. From the perspective of neural efficiency, HbO and HbT patterns instead reflect how optimally cognitive resources are allocated. Experts may display lower PFC activation while achieving high performance, suggesting more efficient neural processing, whereas novices often recruit larger cortical regions to perform at a similar level. Over time, training can restructure these activation patterns, with reduced HbO marking an improved cognitive strategy rather than diminished engagement. Consequently, both increases and decreases in fNIRS signals should be interpreted as markers of adaptive cortical regulation, rather than direct proxies for occupational workload.

Additionally, functional connectivity serves as a robust indicator of occupational workload. Studies have observed increased functional connectivity between the PFC, FPA and motor areas across diverse professions, including seafarers (63, 69), air traffic controllers (92), drivers (41), firefighters (62), and student nurses (77). This heightened connectivity reflects the enhanced integration of cognitive and motor processes essential for executing complex tasks that require simultaneous attention, decision-making, and motor responses (27, 107). These findings are corroborated by neuroimaging studies utilizing fNIRS and EEG, which have demonstrated that increased workload is associated with increased connectivity within executive and attentional networks (22, 108, 109). Thus, it appears that improved functional connectivity underlies the effective integration of cognitive processes that enables adaptive responses to complex task demands and the maintenance of performance under pressure.

Despite the effective use of fNIRS in assessing occupational workload in safety-critical and cognitively demanding professions, research in typical office settings remains scarce (51, 72, 76, 84, 86). This gap is significant, given that office work constitutes a large portion of employment and involves specific cognitive demands. Office tasks often require sustained attention, multitasking, and managing information overload, leading to cognitive fatigue and potential neural overload (110, 111). The included studies demonstrate that even routine office tasks can impose substantial cognitive load, reflected in increased HbO concentrations in the prefrontal cortex (PFC). Midha et al. (51) found elevated HbO in the left PFC during more difficult reading tasks with interruptions. Similarly, Kakehashi et al. (86) reported increased HbO in the PFC during challenging arithmetic and copying tasks. These findings indicate that typical office activities engage executive functions mediated by the PFC. Prolonged high cognitive load can lead to neural overload in the PFC, resulting in cognitive impairment, reduced performance, and increased error rates (31–33). According to Cognitive Load Theory (2, 3), when task demands exceed an individual’s cognitive resources, executive functions such as decision-making and attentional control are impaired.

Understanding the neural correlates of occupational workload in office environments is crucial for developing interventions to prevent cognitive overload and its adverse effects on employee well-being and performance (15, 88). Utilizing fNIRS in these settings can provide valuable insights into how everyday work demands affect cortical activity, informing evidence-based strategies for workload management. Therefore, future research should employ fNIRS to investigate occupational workload in office settings. Such studies are essential to bridge the gap between neuroscience and occupational health promotion, ultimately enhancing employee well-being and productivity.

Our analysis revealed that 26 of the 41 studies used standardized optode placement based on the EEG 10–20 system or its extensions (10-10 and 10-5). Standardized EEG positions facilitate virtual registration of fNIRS optodes and probabilistic estimation of Montreal Neurological Institute (MNI) coordinates (112–114). Combining this method with functional localizers accounts for individual variability and allows for a more detailed determination of optimal placement (115). These practices align with the work of Yücel et al. (116), which emphasizes the importance of standardized placement protocols to increase accuracy and reproducibility. Menant et al. (117) and Pinti et al. (47) also underscore the necessity of precise placement methods to ensure the quality of fNIRS data, highlighting the 10–20 EEG system as the predominant placement strategy. Our findings indicate a common approach among the reviewed studies regarding optode placement strategies, primarily favoring the EEG 10–20 system. However, we observed considerable diversity in the signal processing methods employed. While standardized optode placement contributes to comparability and reproducibility, variations in signal processing techniques may affect the consistency of results across studies. It is crucial to consider the methodological rigor necessary to ensure the accuracy, comparability, and reproducibility of fNIRS research findings. Precise localization of functionally active brain regions remains a central concern in neuroscience research (118, 119). Although fNIRS does not provide direct anatomical information, standardized optode placement is essential for ensuring comparability of results (120). Co-registration with fMRI is considered the gold standard for anatomical localization but is often impractical due to resource constraints (120, 121). Alternatively, researchers can use a digitizer to map the three-dimensional coordinates of the fNIRS channels onto an anatomical atlas (112, 117).

While most studies adopted standardized optode placement, only 17 explicitly implemented methods to correct systemic and extracerebral artifacts. Techniques used included wavelet filters, visual inspections to remove motion artifacts, and the use of reference channels. It is important to note that reference channels must be independent of the target region to avoid influencing the feedback signal (122). Wavelet filters are particularly effective in removing motion artifacts and are considered promising for correcting fNIRS data (120, 121). The use of additional measurements to control subtle movements and systemic factors like heart rate, respiration, or mean arterial pressure was largely neglected, appearing in only one study (77). Future research could incorporate electromyography (EMG) signals as control variables to minimize motion artifacts by including them in the general linear model used for signal calculation (57, 101). Monitoring heart rate through heart rate monitors or electrocardiography can further support the interpretation of cortical hemodynamic changes, as these are associated with systemic blood flow variations (98, 123, 124), cognitive performance, and mental workload (21, 125).

An efficient method for correcting extracerebral physiological signals is the use of short-separation channels combined with the GLM (126–128). Only two (63, 85) of the included studies employed this technique, possibly due to hardware limitations in most fNIRS systems (124, 129, 130). Implementing advanced artifact correction methods is essential to enhance data quality and validity. Careful selection and application of signal processing methods are necessary to avoid distorted or misleading information, especially since there are no established standards for processing fNIRS data (47, 98, 131). Future studies should explore various methods and provide detailed information to facilitate successful replication.

We also observed inconsistencies in sample sizes, with many studies using inadequate participant numbers, limiting the reliability of their findings. Only two study (77, 90) conducted a sensitivity power analysis using G*Power 3 to determine sample size (132). This aligns with trends in neuroimaging research, where statistical evaluations of sample size are rare, leading to low statistical power and higher variability in effect size estimates (133, 134). Small samples tend to overestimate reported effects (135). To address this, researchers advocate for sample size planning based on adequate power analyses to make findings more meaningful (136, 137). Defining the “Smallest Effect Size of Interest” (SESOI) is a potential solution, allowing studies to detect effects that are practically or clinically relevant and enhancing resource efficiency (138, 139). Doing so makes it possible to detect effects that are practically or clinically relevant, thus ensuring more efficient use of resources and strengthening the robustness of the results. Achieving an accurate power analysis also depends on clearly defined hardware configurations, optode placement, and data preprocessing methods (138, 139). Variations in these parameters can alter signal quality and effect sizes, which in turn affects the assumptions underpinning sample-size calculations. Adopting standardized protocols and transparent reporting of these parameters, as recommended in best-practice guidelines (47), will help researchers more reliably estimate the sample size needed for their particular fNIRS setup. Ideally, these details should be established and preregistered before data collection, ensuring methodological consistency and allowing future studies to compare their results across different contexts (136, 137).

A significant issue is the widespread use of quasi-experimental designs, which offer limited insights into intervention effectiveness and measurement validity (140). Transitioning to RCTs is essential to strengthen the evidence base of fNIRS methods. RCTs, regarded as the gold standard in clinical research, provide a robust assessment of causal effects (141). Implementing RCTs would enhance methodological rigor, leading to more reliable and generalizable results (142). The lack of standardized protocols and methodological consensus in fNIRS research hampers comparability and reproducibility (47). RCTs could mitigate these issues by promoting systematic and controlled approaches to investigating intervention effects (24). Standardized protocols would significantly improve data quality and consistency, crucial for establishing fNIRS as a reliable neuroimaging tool in studying occupational workload (143). For example, Yücel et al. (116) emphasized the importance of standardized procedures for enhancing study quality and comparability.

### 4.1 Limitations and strengths

First, the publication bias cannot be entirely excluded, as variations in terminology across studies may have led to the omission of relevant research. Secondly, the review was limited to studies published in English and German up to March 2025, potentially overlooking significant research in other languages. Thirdly, the terminology for the concept of occupational workload is not consistently defined and is used differently across various fields. This lack of terminological consistency complicates the systematic identification and analysis of the phenomenon in the existing literature.

Despite the limitations mentioned, this systematic review provides valuable insights into the application of fNIRS in occupational contexts. The study shows considerable methodological variability, particularly in the choice of signal processing techniques and the use of standardized protocols. The comprehensive data extraction methods enabled detailed analyses of various study characteristics, including occupation type, professional experience, and specific workload types, illustrating the diverse approaches to using fNIRS across different work environments. A unique strength of this review is its focus on methodological approaches for signal processing with fNIRS, specifically in the context of occupational workload. By systematically examining the signal processing techniques used across studies, this review highlights current practices and identifies areas for methodological improvement. This emphasis on signal processing is crucial, as it directly impacts the quality and reliability of fNIRS data. Furthermore, the review underscores the need for standardized protocols and methodological consistency to improve the comparability and reproducibility of fNIRS studies in occupational health research.

## 5 Conclusion

This systematic review demonstrates that fNIRS is an effective tool for assessing occupational workload across various professions. The consistent observation of increased HbO concentrations in the PFC during work-related tasks highlights fNIRS’s capability to detect neural correlates of cognitive demands in real-world settings. In safety-critical and cognitively demanding occupations, such as aviation and maritime navigation, fNIRS has provided valuable insights into the neural mechanisms underlying task performance and workload management. The application of fNIRS in typical office environments remains limited, despite a large portion of employment inherent in office work. The existing studies indicate that even routine office tasks can impose substantial cognitive load, leading to increased PFC activation. Expanding fNIRS research into these settings is crucial for understanding and mitigating cognitive fatigue and neural overload among office workers. This could inform the development of evidence-based interventions aimed at enhancing employee well-being and productivity. To advance the field, methodological improvements are necessary. Standardizing optode placement, employing consistent signal processing techniques, and increasing sample sizes will enhance the validity and comparability of findings. Incorporating randomized controlled trials will further strengthen the evidence base, enabling robust conclusions about fNIRS’s effectiveness in different occupational contexts.

In summary, fNIRS holds significant promise as a non-invasive method for evaluating occupational workload. By bridging neuroscience and occupational health promotion, it can contribute to strategies that promote employee well-being across diverse work environments.

## Data Availability

The original contributions presented in the study are included in the article/Supplementary material, further inquiries can be directed to the corresponding author.
