# Supplementary material for: The application of fNIRS in studies on occupational workload: a systematic review
Source: Front Public Health. 2025 Apr 22;13:1560605. doi: 10.3389/fpubh.2025.1560605 (PMC12053328; doi:10.3389/fpubh.2025.1560605)
Supplement: Supplementary file 1 [file Data_Sheet_1.docx]

Supplementary Material

# Supplementary Data

This document provides supplementary material about the manuscript " The Application of fNIRS in Studies on Occupational Workload: A Systematic Review”

**Details of JBI ratings**

To assess the methodological quality of the included studies we used the revised checklist for quasi-experimental studies of the Joanna Briggs Institute (JBI) critical appraisal tools (Barker et al. 2024). Table S1 shows detailed results of the ratings. Here we further specify the criteria we applied for the ratings.

# *Item 2: Existence of control group*

Within-subject designs were required to be randomized.

## *Item 3: Similar participants in compared groups*

We assumed participants were similar in terms of demographic characteristics and workplace stressors, even if a study did not explicitly report a check for similarity.

## *Item 4: Similar treatment in compared groups*

If a study did not report any other interventions or exposures, we assumed no additional treatment occurred, and that participants in both groups were exposed similarly to external variables. Consequently, this item was rated as "Yes."

## *Item 5: Multiple measurement points of the outcome*

We deviated from the JBI manual, which requires a minimum of four measurement points before and after the intervention. Given the novelty of this research field, we considered two measurement points sufficient (e.g., a standard pre-post design or a time series with multiple points).

## *Item 8: Completion of follow-up*

Studies were not required to include post-intervention follow-up measurements to satisfy this criterion. For within-subject study designs, we assessed whether there were participants who only completed one condition. If a study focused on regulatory performance without at least two intervention sessions on different days, this criterion was marked "Not applicable."

## *Item 9: Appropriate statistical methods*

We rated this item "No" if no power analysis was conducted and the study was not labeled pilot, proof-of-concept/proof-of-principle, or feasibility study.

# Supplementary Figures and Tables

Table S1. Quality assessments of the included studies according to the checklist for quasi-experimental studies of the Joanna Briggs Institute (JBI) critical appraisal tools.

| **First author (Year)** | **1. Cause and effect** | **2. Control condition** | **3. Similar participants** | **4. Similar treatment** | **5. Multiple measures** | **6. Same outcome measures** | **7. Outcome reliability** | **8. Follow-up complete** | **9. Statistical methods** | **Total score** |
| --- | --- | --- | --- | --- | --- | --- | --- | --- | --- | --- |
| Abujelala et al. (2021) | Y | Y | Y | Y | Y | Y | Y | N/A | N | 7 |
| Ahlstrom (2015) | Y | N | N/A | N/A | N | N/A | Y | N/A | N | 2 |
| Aksoy et al. (2025) | Y | N | N/A | N/A | Y | N/A | Y | N/A | N | 3 |
| Alyan et al. (2021) | Y | N | N/A | N/A | N | N/A | Y | N/A | N | 2 |
| Alyan et al. (2021) | Y | N | N/A | N/A | Y | N/A | Y | N/A | N | 3 |
| Andreu-Perez et al. (2012) | Y | N | N/A | N/A | Y | N/A | Y | N/A | N | 3 |
| Ayaz et al. (2011) | Y | N | N/A | N/A | N | N/A | N | N/A | N | 1 |
| Chong et al. (2020) | Y | Y | Y | Y | Y | Y | Y | Y | Y | 9 |
| Fan & Yang (2023) | Y | N | N/A | N/A | Y | N/A | Y | N/A | N | 3 |
| Fan et al. (2020) | Y | N | N/A | N/A | N | N/A | Y | N/A | N | 2 |
| Fan et al. (2025) | Y | N | N/A | N/A | Y | N/A | Y | N/A | N | 3 |
| Harrison et al. (2014) | Y | N | N/A | N/A | Y | N/A | Y | Y | N | 4 |
| Isbilir et al. (2019) | Y | N | N/A | N/A | Y | N/A | Y | N/A | N | 3 |
| Kakashi et al. (2018) | Y | N | N/A | N/A | N | N/A | Y | N/A | N | 2 |
| Kawaguchi et al. (2024) | Y | N | N/A | N/A | N | N/A | Y | N/A | N | 2 |
| Le et al. (2018) | Y | N | N/A | N/A | Y | N/A | Y | N/A | N | 3 |
| Li et al. (2019) | Y | N | N/A | N/A | Y | N/A | Y | N/A | N | 3 |
| Li et al. (2021) | Y | N | N/A | N/A | Y | N/A | Y | Y | N | 4 |
| Li et al. (2024) | Y | Y | Y | N | Y | Y | Y | Y | Y | 8 |
| Liu et al. (2024) | Y | N | N/A | N/A | Y | N/A | Y | N/A | N | 3 |
| Menda et al. (2010) | Y | Y | Y | Y | Y | Y | Y | Y | N | 8 |
| Midha et al. (2020) | Y | N | N/A | N/A | N | N/A | Y | N/A | N | 2 |
| Modi et al. (2017) | Y | N | N/A | N/A | N | N/A | Y | N/A | N | 2 |
| Modi et al. (2019) | Y | N | N/A | N/A | Y | N/A | Y | N/A | N | 3 |
| Mora et al. (2022) | Y | Y | Y | Y | Y | Y | Y | Y | N | 8 |
| Naik et al. (2024) | Y | N | N/A | N/A | Y | N/A | Y | N/A | N | 3 |
| Palzes et al. (2019) | Y | Y | Y | Y | Y | Y | Y | N/A | Y | 8 |
| Pooladvand & Hasanzadeh (2022) | Y | N | N/A | N/A | Y | N/A | Y | N/A | N | 3 |
| Pooladvand et al. (2024) | Y | N | N/A | N/A | Y | N/A | Y | N/A | N | 3 |
| Singh et al. (2018) | Y | N | N/A | N/A | N | Y | Y | N/A | N | 3 |
| Sun et al. (2020) | Y | N | N/A | N/A | N | N/A | Y | N/A | N | 2 |
| Tang et al. (2022) | Y | N | N/A | N/A | Y | N/A | Y | N/A | N | 3 |
| Tian et al. (2022a) | Y | N | N/A | N/A | Y | N/A | Y | N/A | Y | 4 |
| Tian et al. (2022b) | Y | N | N/A | N/A | N | N/A | Y | N/A | N | 2 |
| Tian et al. (2024) | Y | N | N/A | N/A | N | N/A | Y | N/A | N | 2 |
| Tyagi et al. (2021) | Y | Y | Y | Y | Y | Y | Y | N/A | N | 7 |
| Verdière et al. (2018) | Y | N | N/A | N/A | Y | N/A | Y | N/A | N | 3 |
| Xu et al. (2019) | Y | N | N/A | N/A | N | N/A | Y | N/A | N | 2 |
| Zhang et al. (2023) | Y | N | N/A | N/A | N | N/A | Y | N/A | N | 2 |
| Zheng et al. (2018) | Y | N | N/A | N/A | Y | N/A | Y | N/A | N | 3 |
| Zheng et al. (2020) | Y | N | N/A | N/A | Y | N/A | Y | N/A | N | 3 |
| Total | 41 | 7 | 7 | 6 | 27 | 8 | 40 | 6 | 4 | M = 3.6  SD = 2,09 |

Y. Yes; N. No; U. Unclear. N/A. Not Applicable; note that in some cases we deviated from the JBI manual (see Supplementary Material for details).

**Table S2:** Studies published from December 2023 to March 2025 fulfilling the inclusion criteria for the present review.

| **Author(s)** | **Year** | **Titel** | **Journal** | **Occupation** |
| --- | --- | --- | --- | --- |
| Aksoy et al. | 2025 | Comparing Behavioral and Neural Activity Changes During Laparoscopic and Robotic Surgery Trainings | Journal of Surgical Education (Vol. 82, Issue 5, May 2025) | surgery residents |
| Fan et al. | 2025 | Towards advanced decision-making support for shipping safety: A functional connectivity analysis | Transportation Research Part E: Logistics and Transportation Review (Vol. 193, January 2025) | watchkeeping officers |
| Kawaguchi et al. | 2024 | Effects of age and flight experience on prefrontal cortex activity in airline pilots: An fNIRS study | Heliyon (Vol. 10, Issue 9, May 2024) | airline pilot captains |
| Li et al. | 2024 | Effect of Mindfulness Training on Skill Performance in Simulator-Based Knee Arthroscopy Training for Novice Residents-A Randomized Controlled Study. | Journal of Surgical Education (Vol. 81, Issue 12, December 2024) | orthopedic residents |
| Liu et al. | 2024 | Distinction of mental health between salesman and R&D in high-tech enterprise: a fNIRS study. | Scientific Reports (Vol.14, 2024) | sales personnel and research and development |
| Naik et al. | 2024 | Novel multimodal sensing and machine learning strategies to classify cognitive workload in laparoscopic surgery. | European Journal of Surgical Oncology (October 2024) | surgeon |
| Pooladvand et al. | 2024 | Identifying at-risk workers using fNIRS-based mental load classification: A mixed reality study | Automation in Construction  (Vol. 164, August 2024) | construction workers |
| Tian et al. | 2024 | The neurocognitive mechanism linking temperature and humidity with miners' working memory: an fNIRS study. | Front Hum Neurosci. (Vol. 18, September 2024) | coalmine workers |
